# Genetic deletion of genes in the cerebellar rhombic lip lineage can stimulate compensation through adaptive reprogramming of ventricular zone-derived progenitors

**DOI:** 10.1186/s13064-019-0128-y

**Published:** 2019-02-14

**Authors:** Alexandre Wojcinski, Morgane Morabito, Andrew K. Lawton, Daniel N. Stephen, Alexandra L. Joyner

**Affiliations:** 1 Developmental Biology Program, Sloan Kettering Institute, 1275 York Avenue, Box 511, New York, NY 10065 USA; 2 Biochemistry, Cell and Molecular Biology Program, Weill Cornell Graduate School of Medical Sciences, New York, NY 10065 USA

**Keywords:** Cerebellum, SHH signaling, GLI2, Nestin-expressing progenitors, Neurogenesis, *Atoh1-Cre*, Regeneration

## Abstract

**Background:**

The cerebellum is a foliated posterior brain structure involved in coordination of motor movements and cognition. The cerebellum undergoes rapid growth postnataly due to Sonic Hedgehog (SHH) signaling-dependent proliferation of ATOH1+ granule cell precursors (GCPs) in the external granule cell layer (EGL), a key step for generating cerebellar foliation and the correct number of granule cells. Due to its late development, the cerebellum is particularly vulnerable to injury from preterm birth and stress around birth. We recently uncovered an intrinsic capacity of the developing cerebellum to replenish ablated GCPs via adaptive reprogramming of Nestin-expressing progenitors (NEPs). However, whether this compensation mechanism occurs in mouse mutants affecting the developing cerebellum and could lead to mis-interpretation of phenotypes was not known.

**Methods:**

We used two different approaches to remove the main SHH signaling activator GLI2 in GCPs: 1) Our mosaic mutant analysis with spatial and temporal control of recombination (MASTR) technique to delete *Gli2* in a small subset of GCPs; 2) An *Atoh1-Cre* transgene to delete *Gli2* in most of the EGL. Genetic Inducible Fate Mapping (GIFM) and live imaging were used to analyze the behavior of NEPs after *Gli2* deletion.

**Results:**

Mosaic analysis demonstrated that SHH-GLI2 signaling is critical for generating the correct pool of granule cells by maintaining GCPs in an undifferentiated proliferative state and promoting their survival. Despite this, inactivation of *GLI2* in a large proportion of GCPs in the embryo did not lead to the expected dramatic reduction in the size of the adult cerebellum. GIFM uncovered that NEPs do indeed replenish GCPs in *Gli2* conditional mutants, and then expand and partially restore the production of granule cells. Furthermore, the SHH signaling-dependent NEP compensation requires *Gli2*, demonstrating that the activator side of the pathway is involved.

**Conclusion:**

We demonstrate that a mouse conditional mutation that results in loss of SHH signaling in GCPs is not sufficient to induce long term severe cerebellum hypoplasia. The ability of the neonatal cerebellum to regenerate after loss of cells via a response by NEPs must therefore be considered when interpreting the phenotypes of *Atoh1-Cre* conditional mutants affecting GCPs.

**Electronic supplementary material:**

The online version of this article (10.1186/s13064-019-0128-y) contains supplementary material, which is available to authorized users.

## Background

The cerebellum (CB) consists of 80% of the neurons in the human brain [1] (60% in mouse [2]), and is involved in balance and motor coordination, but also modulates language, reasoning and social processes via its connections throughout the forebrain [3–7]. The CB undergoes its major growth in the third trimester and infant stage in humans, and the first 2 weeks after birth in mice, primarily due to expansion of the granule cell precursor (GCP) pool in the external granule cell layer (EGL) [8–10]. Given the late development of the CB compared to other brain regions, the CB is particularly sensitive to environmental and clinical factors that impact on growth (or cause injury) around birth. Furthermore, CB hypoplasia and prenatal injury is the second leading factor associated with autism [11]. It is therefore important to identify genes that regulate cerebellum development. Many of the genes have been identified based on motor defects in homozygous null mutant mice, or in conditional mutants that remove genes in specific cell lineages. Intrinsic growth compensation mechanisms involving lineages where the gene does not function could however, obscure the normal function of a gene in cerebellar growth.

The CB develops from two germinal zones. The ventricular zone (VZ) gives rise to all the inhibitory neurons, including Purkinje cells (PCs) [12] as well as Nestin-expressing progenitors (NEPs) that expand in the cerebellar cortex after birth to produce astrocytes, including specialized Bergmann glia, and late born interneurons of the molecular layer [13, 14]. *Ptf1a*^*Cre*^ mice have been used to delete genes in inhibitory neurons and some glia [15]. Excitatory neurons including granule cells (GCs) originate from the upper rhombic lip [16–18]. In mice, the EGL is established between embryonic day (E) 13.5 and E15.5. *Atoh1*-expressing GCPs then proliferate and expand in the EGL until ~postnatal day (P) 15 in response to Sonic Hedgehog (SHH) secreted by PCs [19–21]. When GCPs become postmitotic, they migrate down Bergmann glial fibers to form the internal granule cell layer (IGL). Interestingly, in rodent models the developing CB has been found to have a remarkable ability to recover from some injuries [22–24]. Indeed, we recently found that proliferating cerebellar GCPs can be replaced via adaptive reprogramming of NEPs after an acute depletion of the perinatal EGL due to irradiation [25–27]. Thus, NEPs in the neonatal CB have highly plastic behaviors. However, whether NEPs are harnessed to replenish cells lost in developmental mutants that lack key factors required for expansion and survival of GCPs has not been addressed.

One of the major pathways driving CB development is HH signaling. There are three hedgehog (*Hh*) genes in mammals, *Indian* (*Ihh*), *Desert* (*Dhh*) and *Shh* [28, 29]. *Shh,* the most widely expressed *Hh* gene, is required for development of most organs [29] by regulating a variety of cell behaviors including cell death, proliferation, specification and axon guidance. The cellular context (i.e. tissue, developmental stage, convergence of other signaling pathways) and concentration of SHH are thought to determine the particular response of a cell to SHH. HH signal transduction is mediated by the receptors Patched1 (PTCH1) and Smoothened (SMO) [28–30]. In the absence of HH signaling, PTCH1 constitutively represses SMO activity, whereas HH binding relieves this inhibition, in part by allowing accumulation of SMO in cilia [31]. The GLI/Ci transcription factors are the effectors of the HH pathway. In mammals, the transcriptional activator (A) and repressor (R) functions of the GLIs have been divided between the three proteins [32]. A general rule is that high levels of HH signaling induce the formation of a GLI2 activator (GLI2^A^) and this leads to transcription and translation of an addition activator, GLI1^A^, while a reduction or absence of the ligand allows for the formation of a GLI3 repressor (GLI3^R^). Importantly, we demonstrated that *Gli1* expression is dependent on GLI2/3^A^, and thus is only expressed in cells receiving a high level of HH signaling [33, 34]. The three *Gli* genes, *Shh*, *Smo*, *Ptch1* and *Ptch2* are expressed in the CB and all but *Ptch2* are required for CB development [20, 21, 35–37]. In particular, we have shown that SHH functions by inducing GLI1^A^/2^A^ and is required for expansion of GCPs, primarily after birth [20, 38], whereas Gli3 is not required in the cerebellum after E12.5 [36]. In addition to the crucial role of SHH in generating the pool of GCs, expansion of NEPs and thus production of NEP-derived interneurons and astroglia (astrocytes and Bergmann glia) also require SHH-signaling [13, 25, 39]. Furthermore, HH-signaling in NEPs is crucial for expansion of NEPs, recovery of the EGL and scaling of interneuron numbers after injury to the EGL [25].

Here we report that deletion of *Gli2* in the vast majority of the GCPs is not sufficient to induce major cerebellar hypoplasia. Using our MASTR technique [40] in a mosaic mutant analysis of the effect of deleting *Gli2* in scattered GCPs, we found that HH/GLI2-signaling is indeed necessary to maintain GCPs in an undifferentiated and proliferative state and to promote their survival. However, and similar to when the EGL is depleted using irradiation, we uncovered that NEPs are harnessed to repopulate the EGL and then wild type progenitors differentiate into GCs when *Gli2* is deleted in most GCPs using an *Atoh1*-driven constitutive Cre [41]. Our results not only provide more evidence for the unusual ability of the CB to recover from perinatal stress, but also reveal that NEP-dependent compensation should be taken into account when studying genes implicated in GCP development or survival and when using the *Atoh1-Cre* transgene.

## Methods

### Animals

The following mouse lines were used: *Gli2*^*flox/flox*^ [20], *Atoh1-Cre* [41], *Atoh-FlpoER, Nestin-FlpoER* (a transgene similar to that described in [40]) and *Rosa26*^*MASTR(frt-STOP-frt-GFPcre)*^ [40], *Atoh1-GFP* [42], *Nes-CFP* [43], *Rosa26*^*FRT-STOP-FRT-TDTom*^ *(Jackson Laboratory, 021875).* The *Atoh-FlpoER* line, was made using the FLPoER1 cDNA described in [40] by subcloning it into the *Atoh1* expression construct described in [17]. All mouse lines were maintained on an outbred Swiss Webster background and both sexes were used for the analysis. Animals were housed on a 12 h light/dark cycle and were given access to food and water ad libitum. All experiments were performed using mice ages P0–P30.

Tamoxifen (Tm, Sigma-Aldrich) was dissolved in corn oil (Sigma-Aldrich) at 20 mg/ml. P2 *Atoh1-FlpoER/+; R26*^*MASTR/+*^; *Gli2*^*flox/flox*^*, Atoh1-FlpoER/+; R26*^*MASTR/+*^; *Gli2*^*flox/flox*^ and P0 *Nes-FlpoER/+; R26*^*FSF-TDTom/+*^, *Nes-FlpoER/+; R26*^*FSF-TDTom/+*^*; Atoh-GFP/+* mice as well as *Nestin-FlpoER/+; R26*^*MASTR/+*^; *Gli2*^*flox/flox*^, *Atoh1-Cre/+; Gli2*^*flox/flox*^ and *Nestin-FlpoER; R26*^*MASTR/+*^; *Atoh1-Cre/+; Gli2*^*flox/flox*^ mice and littermate controls received one 200 μg/g dose of Tm via subcutaneous injection. 50 μg/g 5-ethynyl-2_-deoxyuridine (EdU; Invitrogen) was administered via intraperitoneal injection (10 mg/ml in sterile saline) one hour before the animals were sacrificed.

### Tissue processing, immunohistochemistry (IHC) and transcript detection

For animals younger than P4, they were anaesthetized by cooling and brains were dissected out and fixed in 4% paraformaldehyde overnight at 4 °C. Animals P4–30 received 50 μl intraperitoneal injections of ketamine and received ice-cold PBS via transcardial perfusion followed by 4% paraformaldehyde. Brains were collected and submersion fixed in 4% paraformaldehyde overnight at 4 °C. Tissues were processed for frozen embedding in optimal cutting temperature (OCT) compound and sectioned in the parasagittal plane on a Leica cryostat at 12 μm. For IHC, sections were incubated overnight at 4 °C with the following primary antibodies: rabbit anti-Ki67 (Thermo Scientific, RM-9106-S0), mouse anti-P27 (BD Pharmigen, 610,241), rabbit anti-PAX6 (Millipore, AB2237), goat anti-GLI2 (R&D System, AF3635), Goat anti-SOX2 (R&D System, AF2018), rabbit anti-GFP (Life Technologies, A11122), rat anti-GFP (Nacalai Tesque, 04404–84), mouse anti-NeuN (Millipore, MAB377) diluted in PBS with 5% BSA (Sigma-Aldrich) and 0.3% Triton X-100 (Fisher Scientific). Sections were then exposed for 2 h at room temperature to secondary species-specific antibodies conjugated with the appropriate Alexa Fluor (1:500; Invitrogen). EdU was detected using a commercial kit (Life Technologies) after the IHC reactions. TUNEL staining and in situ hybridization were performed according to standard protocols. *Cre* and *Gli1* cDNAs were used as the template for synthesizing digoxygenin-labeled riboprobes. Images were collected on a DM6000 Leica microscope and processed using Photoshop software.

### Live imaging

Ex vivo cerebellar slice culture was done as previously described [25]. Briefly, P8 cerebella were embedded in 2.5% low-melting point agarose and saggitally sliced at 250 μM on a Vibratome. Slices were immediately taken to either a Leica TCS SP8 or SP5 confocal microscope platform. Slices were maintained in Eagle’s Basal Medium with 2 mM L-glutamine, 0.5% glucose, 50 U/ml Penicillin-streptomycin, 1xB27 and 1xN2 supplements at 37 °C and 5% CO_2_. Image stacks were acquired every 5 min for ~ 4 h. Cell tracking was performed using Imaris software. The autoregressive tracking function was employed with a spot size of 6 μM and a step size of 7 μM. Manual correction was performed.

### Quantifications and statistical analyses

ImageJ software was used to measure the area (mm^2^) of cerebellar sections near the midline. For all IHC staining, cell counts were obtained using ImageJ and Neurolucida Software. For each developmental stage, three sections were analyzed per animal and ≥ 3 animals. Statistical analyses were performed using Prism software (GraphPad) and significance was determined at *P* < 0.05. All statistical analyses were two-tailed. For two-group comparisons with equal variance as determined by the *F*-test, an unpaired Student’s *t* test was used. Welch’s correction was used for unpaired *t*-tests of normally distributed data with unequal variance. *P* values are indicated in the figures. No statistical methods were used to predetermine the sample size, but our sample sizes are similar to those generally employed in the field. No randomization was used. Data collection and analysis were not performed blind to the conditions of the experiments.

## Results

### Mosaic analysis reveals SHH-GLI2 signaling is critical for maintaining GCPs in an undifferentiated proliferative state and promoting their survival

**Fig. 1:**
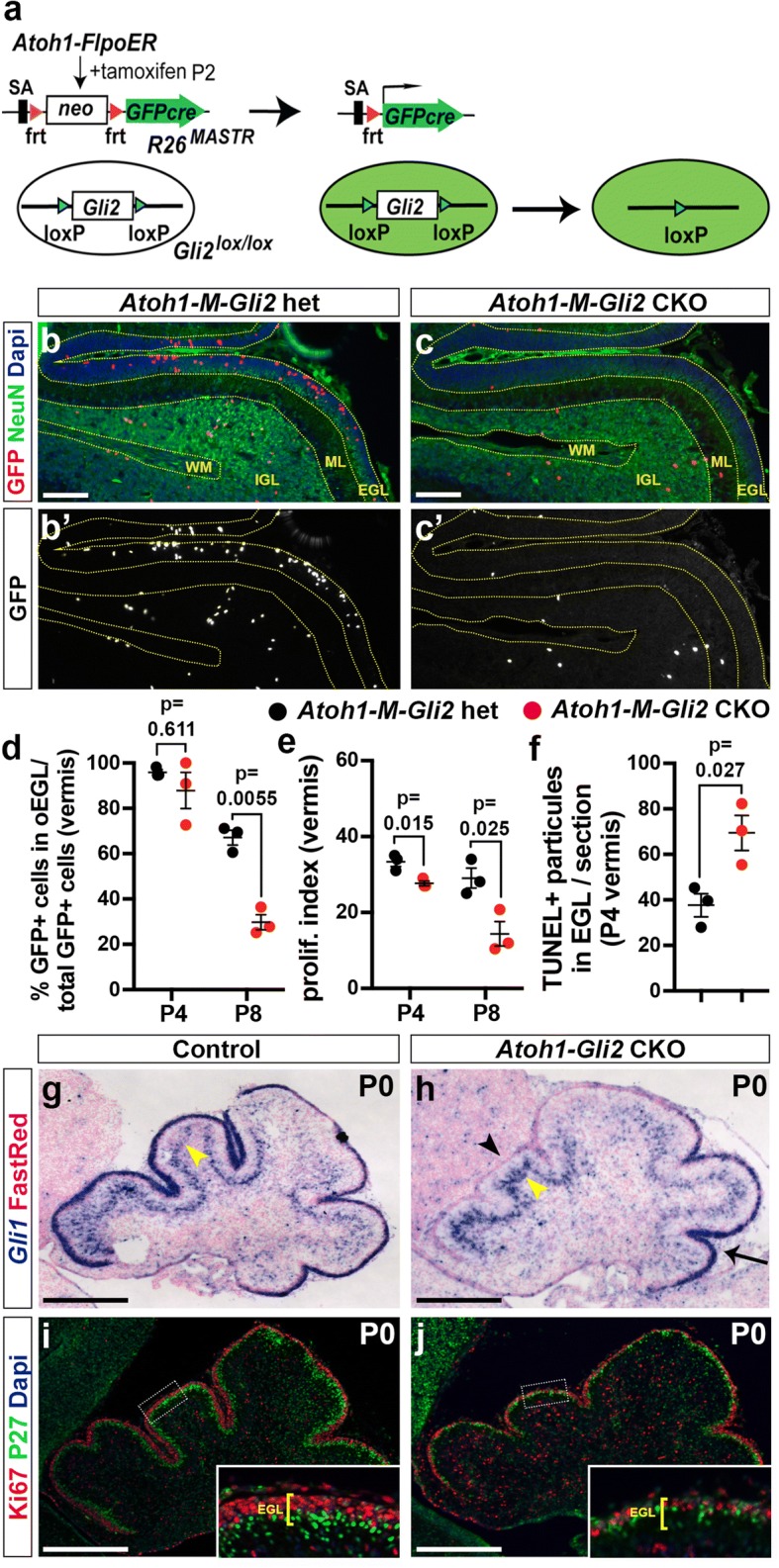
HH-GLI2 signaling maintains GCP in an undifferentiated state and promotes their survival. **a** Schematic representation of the MASTR approach. The *R26*^*MASTR*^ allele expresses a GFPcre fusion upon Flp induced deletion of a *neo* (STOP) cassette. When the *R26*^*MASTR*^ allele and the *Atoh1-FlpoER* transgene are combined with a floxed gene such as *Gli2*, recombination of the loxP sites occurs in > 98% of GFP+ cells within 3 days of administrating tamoxifen (Tm) at P2. The mutant cells and their progeny can subsequently be identified by the continuous expression of eGFP from the *R26* allele. **b**-**c** Fluorescent Immuno-Histo-Chemical (FIHC) detection of the indicated proteins and dapi on mid-sagittal sections (lobule VII and VIII) of P8 control *R26*^*MASTR/+*^; *Atoh1-FlpoER/+; Gli2*^*lox/+*^ (*Atoh1-M-Gli2* het, **b**) and *R26*^*MASTR/+*^; *Atoh1-FlpoER/+; Gli2*^*lox/lox*^ (*Atoh1-M-Gli2* CKO, **c**) mice treated Tm at P2. **d**-**f** Graphs of the proportion of GFP+ cells in the outer (o) EGL at P8 (*n* = 3) (**d**), the proliferation index at P8 (% [GFP+ EdU+] cells of all [GFP+] cells in the oEGL) (*n* = 3) (**e**) and the number TUNEL+ particles per section at P4 (*n* = 3) (**f**) in *Atoh1-M-Gli2* het (control, black) and *R26*^*MASTR/+*^; *Atoh1-FlpoER/+; Gli2*^*lox/lox*^ (*Atoh1-M-Gli2* CKO, red) mice treated with Tm at P2. All of the analyses were performed on 3 midline sections per brain. All graphical data are presented as means ± SEM and significance determined using two-tailed T-test. **g**-**h** In situ hybridization of *Gli1* mRNA on P0 mid-sagittal cerebellar sections of *Gli2*^*lox/lox*^ (control, **g**) and *Atoh1-Cre/+; Gli2*^*lox/lox*^ (*Atoh1-Gli2* CKO, **h**) mice. Black arrowhead indicates the loss of *Gli1* expression in the anterior mutant EGL, black arrow *Gli1* remaining in the posterior EGL and yellow arrowheads indicate *Gli1* expression in Bergmann glia in the Purkinje Cell Layer (PCL). **i**-**j** FIHC detection of the indicated proteins and dapi on P0 mid-sagittal cerebellar sections of *Gli2*^*lox/lox*^ (control, **i**) and *Atoh1-Cre/+; Gli2*^*lox/lox*^ (*Atoh1-Gli2* CKO, **j**) mice. High power images are shown of the areas indicated by white rectangles and the thickness of the EGL is indicated by yellow bracket. Scale bars represent 100 μm (**b**-**c**) and 500 μm (**g**-**j**)

Our previous studies demonstrated that loss of the majority of HH-signaling in the entire CB at mid-gestation (*Nes-Gli2* conditional knockout or CKO - *Nestin-Cre; Gli2*^*flox/flox*^ mice) results in an almost complete lack of GCPs at birth and a very diminished CB in adults [20]. Since HH-signaling is required after birth in NEPs for their expansion and production of late born interneurons and astrocytes in the CB [13], it is possible that part of the phenotype observed in *Nes-Gli2* CKOs was due to loss of HH-signaling in Non-GCP cells. We therefore took two approaches to test the cell autonomous requirement for HH signaling in GCPs. First we used the *R26*^*FSF-GFPcre*^ MASTR allele (*R26*^*MASTR*^) [40] and a *Atoh1-FlpoER* transgene to knock out *Gli2* in scattered GCPs at ~P3 by administering Tamoxifen (Tm) at P2 and analyzed the percentage of undifferentiated GFP+ GCPs (GFP+ cells in the proliferating outer EGL/total GFP+ cells – proliferating and post mitotic) at both P4 and P8 (Fig. 1a-c). We did indeed observe a significant decrease in the percentage of GFP+ cells that were GCPs in the medial CB (vermis) of P8 *Atoh1-M-Gli2* CKOs (*R26*^*MASTR/+*^; *Atoh1-FlpoER/+; Gli2*^*flox/flox*^ mice; *n* = 3;) compared to *Atoh1-M-Gli2* heterozgous (het) controls (*R26*^*MASTR/+*^; *Atoh1-FlpoER/+; Gli2*^*flox/+*^ mice; *n* = 3) (29.79% compared to 67.09%) (Fig. 1d). Using a 1 h pulse of EdU, we found that the proliferation index (#EdU+ GFP+ cells in the outer EGL/total GFP+ cells in the outer EGL) of *Atoh1-M-Gli2* CKO GCPs was significantly decreased compared to controls (*n* = 3; 14.39% compared to 29.06%) (Fig. 1e). At P4, there was no significant difference in the percentage of undifferentiated GFP+ GCPs between *Atoh1-M-Gli2* CKOs and controls (*p* = 0.162) (Fig. 1d). However, we observed a significant decrease in the proliferation index in P4 *Atoh1-M-Gli2* CKO cerebella (CKO vs control, *p* = 0.015) (Fig. 1e). At P4, only 2 days after Tm injection, the number of GFP+ cells in the oEGL was not significantly reduced (CKO vs control, *p* = 0.081) (Additional file 1: Figure S1a). Nevertheless, TUNEL staining revealed a significant increase in cell death in the entire EGL at P4 (69.44 ± 7.76/section in mutants vs 37.67 ± 5.1 in controls, *p* = 0.027). We performed the same analysis in the lateral CB (hemispheres) and found a similar outcome (Additional file 1: Figure S1b-d). These results reveal that HH-signaling through GLI2 plays an important role in maintaining GCPs in an undifferentiated state, and also promotes their proliferation and survival.

As an alternative approach to a mosaic mutant analysis, we deleted *Gli2* in the vast majority of GCPs (*Atoh1-Cre/+; Gli2*^*flox/flox*^ or *Atoh1-Gli2* CKOs). Consistent with previous studies using whole cerebellum *Cre* transgenes and our mosaic analysis, at P0 the anterior vermis of *Atoh1-Gli2* CKOs (*n* = 5) appeared consistently smaller than controls (*Gli2*^*flox/flox*^) and the EGL was greatly diminished (Fig. 1g-j). SHH-GLI2 signaling loss was confirmed by the lack of *Gli1* expression in the EGL of *Atoh1-Gli2* CKO cerebella (Fig. 1g-h). Moreover, proliferation (Ki67) in the outer EGL and differentiation (P27 marking post mitotic cells in the inner EGL) were disrupted in the mutant EGL since two distinct EGL layers were not present (Fig. 1i-j). Interestingly, we observed an apparent increase of *Gli1* expression in the Purkinje cell layer (PCL) suggesting that deletion of *Gli2* in the EGL induced a cell non-autonomous up-regulation of HH-signaling in this layer (star in Fig. 1g-h). The lack of a phenotype in the posterior vermis is likely explained by low expression of *Cre* [44] in this region and thus low recombination [45] (Additional file 2: Figure S2).

All together, these results confirm a major role played by SHH-signaling through GLI2 to promote the expansion of the EGL and thus ensure the generation of the correct number of GCs.

### The size of the *Atoh1-Gli2* CKO cerebellum progressively recovers after birth

**Fig. 2:**
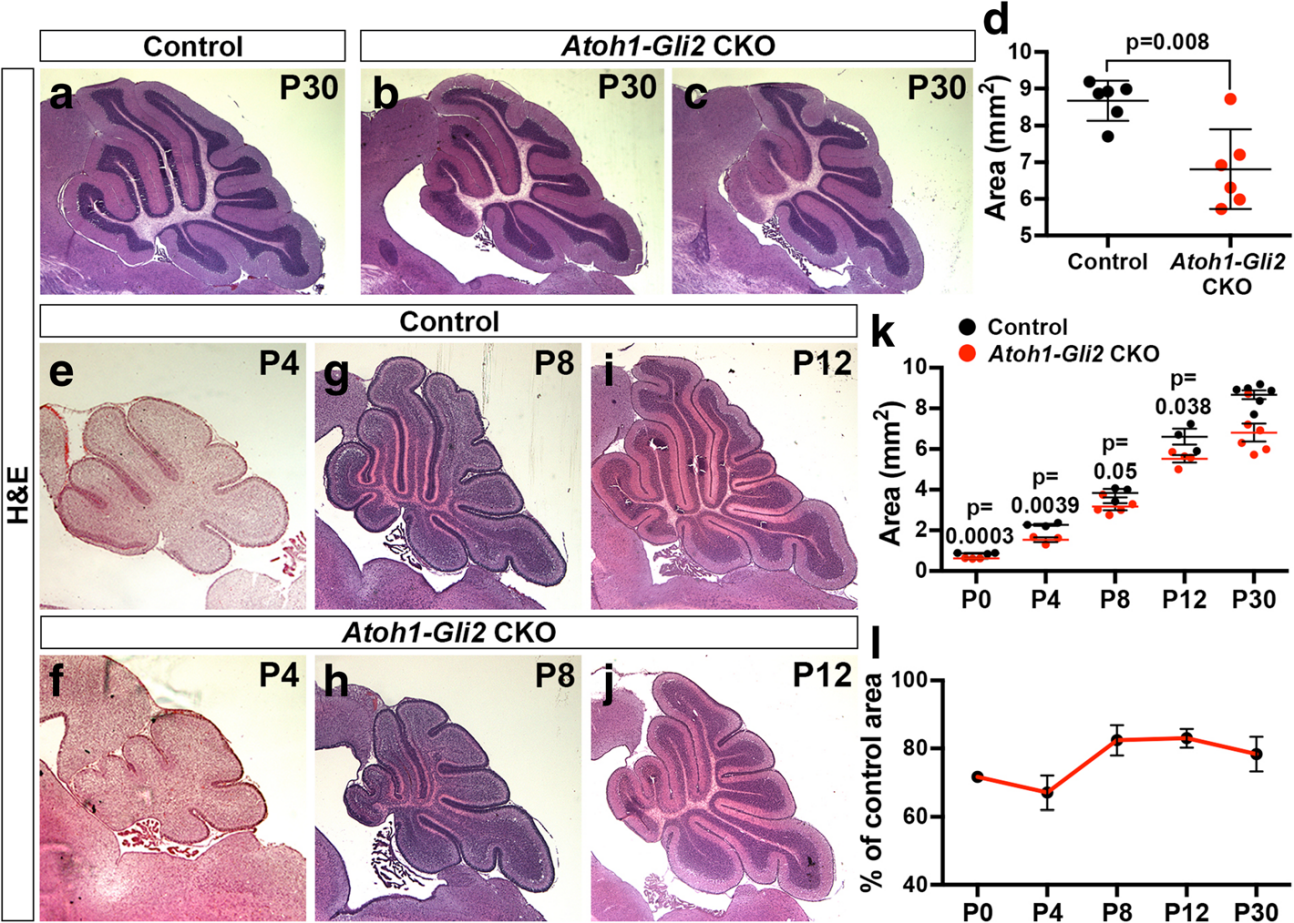
The size of the cerebellum partially recovers in *Atoh-Gli2* CKOs over time. **a**-**c** Mid-sagittal sections of P30 *Gli2*^*lox/lox*^ (control, **a**) and *Atoh1-Cre/+; Gli2*^*lox/lox*^ (*Atoh1-Gli2* CKO, **b**-**c**) cerebella stained with Hematoxilin and Eosin (H&E). **d** Graph of the area of mid-sagital CB sections of P30 *Gli2*^*lox/lox*^ (control, black) (*n* = 6) and *Atoh1-Cre/+; Gli2*^*lox/lox*^ (*Atoh1-Gli2* CKO, red) (*n* = 6) mice. **e**-**j** Mid-sagittal CB sections of P4 (**e**-**f**), P8 (**g**-**h**) and P12 (**i**-**j**) *Gli2*^*lox/lox*^ (control, **e**, **g** and **i**) and *Atoh1-Cre/+; Gli2*^*lox/lox*^ (*Atoh1-Gli2* CKO, **f**, **h** and **j**) mice stained with H&E. **k** Graph of the area of 3 mid-sagital sections of *Gli2*^*lox/lox*^ (control, P0: *n* = 3, P4: *n* = 3, P8: *n* = 3, P12: *n* = 3 and P30: *n* = 6) and *Atoh1-Cre/+; Gli2*^*lox/lox*^ (*Atoh1-Gli2* CKO, P0: *n* = 3, P4: *n* = 3, P8: *n* = 6, P12: *n* = 4 and P30: *n* = 6) cerebella. **l** Graph showing the decrease in area of 3 mid-sagital sections of *Atoh1-Gli2* CKO cerebella as a percentage of controls during development. All graphical data are presented as means ± SEM and significance determined using two-tailed T-test. Scale bars represent 1 mm

**Fig. 3:**
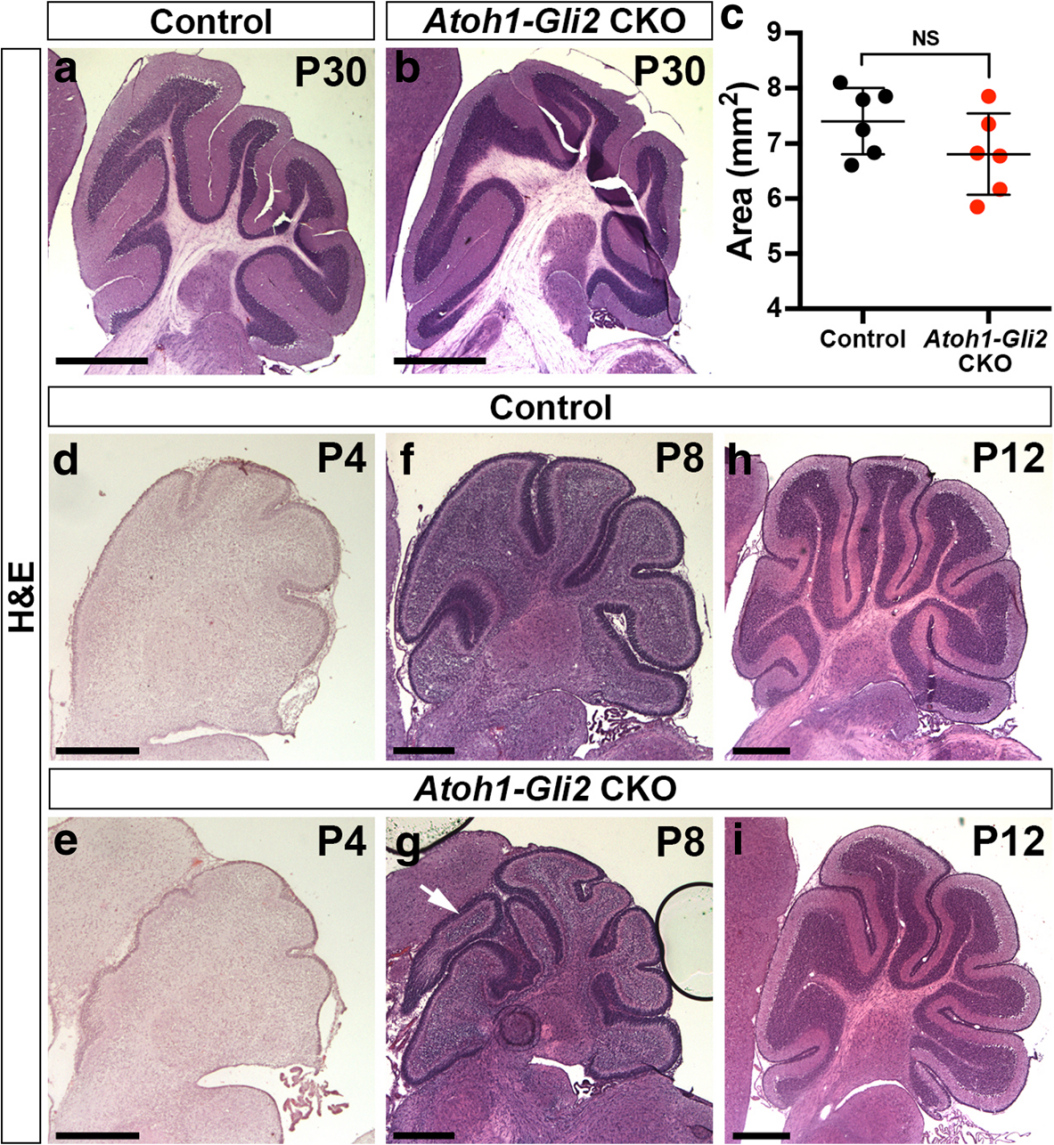
Hemispheres recover better than the vermis in *Atoh1-Gli2* CKO mice. **a**-**b** Hemispheric sagittal sections of P30 *Gli2*^*lox/lox*^ (control, **a**) and *Atoh1-Cre/+; Gli2*^*lox/lox*^ (*Atoh1-Gli2* CKO, **b**) cerebella stained with Hematoxilin/Eosin (H&E). **c** Graph of the area of hemispheric sagital sections of P30 *Gli2*^*lox/lox*^ (control, black) (*n* = 6) and *Atoh1-Cre/+; Gli2*^*lox/lox*^ (*Atoh1-Gli2* CKO, red) (*n* = 6) CB. (**d**-**i**) Hemispheric sagittal sections of P4 (**d**-**e**), P8 (**f**-**g**) and P12 (**h**-**i**) *Gli2*^*lox/lox*^ (control, **d**, **f** and **h**) and *Atoh1-Cre/+; Gli2*^*lox/lox*^ (*Atoh1-Gli2* CKO, **e**, **g** and **i**) cerebella stained with H&E. White arrow indicates the presence of extra folia. Scale bars represent 1 mm (**a**-**b**) and 500 μm (**b**-**c** and **d**-**i**)

We have recently shown that the size of the CB can recover to ~ 80% its normal size after postnatal injury (irradiation) to the EGL [25]. To test whether genetic ablation of *Gli2* in the EGL can trigger a similar recovery mechanism, we analyzed the phenotype of adult (P30) *Atoh1-Gli2* CKO cerebella. The area of mid-sagittal sections of P30 animals was quantified, and revealed only a 21.7 ± 12.0% reduction (*n* = 6) in *Atoh-Gli2* CKOs compared to littermate controls (Fig. 2a-d). Unlike normal mice, we observed a large variability in the area of the midline cerebellum and degree to which the IGL was depleted in mutants, indicating some mutant cerebella recovered the EGL better than others (compare Fig. 2b and c). We then measured the area of midsagittal cerebellar sections from P4, P8 and P12 mice to determine the growth trajectory of mutant mice compared to controls (Fig. 2e-j). The size of *Atoh1-Gli2* CKOs cerebella was significantly reduced by ~ 20% at all time points (Fig. 2k-l). Since our mosaic mutant results showed a similar behavior of *Gli2* CKO GCPs in the hemispheres and vermis, we analyzed the phenotype of *Atoh1-Gli2* CKOs in the hemisphere. Curiously, unlike the vermis we did not observe a significant decrease in the size of the mutant hemispheres at P30 compared to controls (*p* = 0.152) (Fig. 3a-c). Analysis of hemispheric sagittal sections from P4 (*n* = 3), P8 (*n* = 3) and P12 (*n* = 3) revealed how the cytoarchitecture recovered (Fig. 3d-i). Interestingly, whereas the vermis of *Atoh1-Gli2* CKOs mice at P8 showed a clear hypoplasia phenotype, the hemispheres exhibited extra folia (arrow in Fig. 3g), suggesting different compensation responses in the two locations of the CB in *Atoh1*-*Gli2* CKOs.

In summary, we found that depletion of the EGL at P0 by removing *Gli2* from embryonic GCPs is not sufficient to induce consistent major hypoplasia of the vermis at P30. This raised the possibility of a compensation mechanism that allows partial recovery of the developing CB after genetic depletion of the EGL.

### Wild type cells replenish the anterior EGL of *Atoh1*-*Gli2* CKOs

**Fig. 4:**
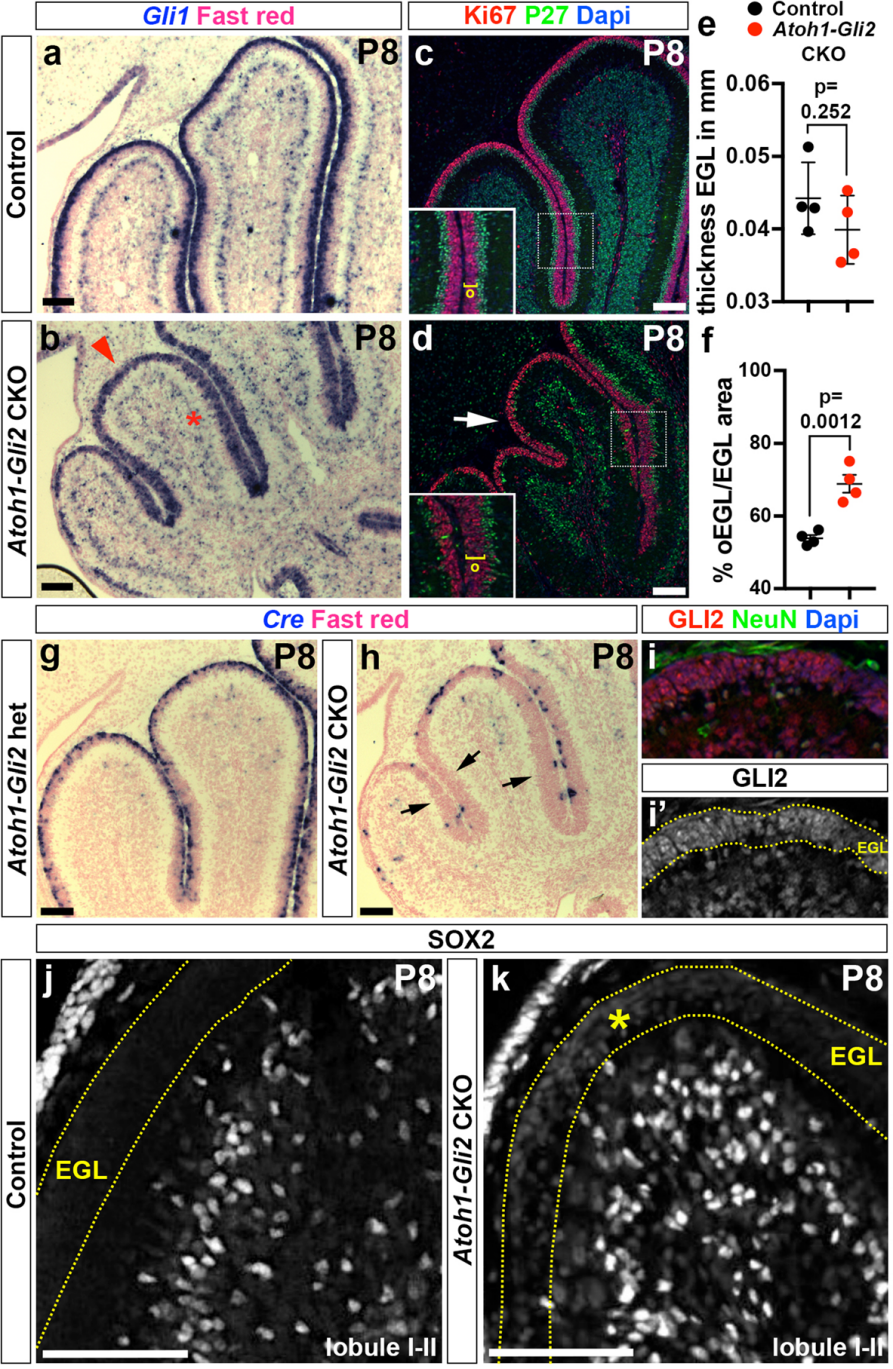
Loss of *Gli2* mutant GCPs at P0 is compensated by wild type (WT) cells at P8. **a**-**b** In situ RNA hybridization analysis of *Gli1* on P8 midsagittal cerebellar sections of *Gli2*^*lox/lox*^ (control, **a**) and *Atoh1-Cre/+; Gli2*^*lox/lox*^ (*Atoh1-Gli2* CKO, **b**) mice. Red arrowhead indicates the strong *Gli1* expression in the mutant EGL and red asterisks indicate normal *Gli1* expression in Bergmann glia in the Purkinje Cell Layer (PCL). **c**-**d** FIHC detection of the indicated proteins and dapi on P8 mid-sagittal cerebellar sections of *Gli2*^*lox/lox*^ (control, **c**) and *Atoh1-Cre/+; Gli2*^*lox/lox*^ (*Atoh1-Gli2* CKO, **b**) mice. High power images are shown of the areas indicated by white rectangles in (**c** and **d**) with the thickness of the outer EGL (o) indicated by yellow brackets. The white arrow in (**b**) indicates the proliferating EGL. **e** and **f** Graphs of the thickness proportion of the EGL at P8 (*n* = 4) (**e**) and the proportion of oEGL area at P8 (% [oEGL] area of total [EGL] area) (*n* = 4) (**f**) in *Gli2*^*lox/lox*^ (CTL, black) and *Atoh1-Cre/+; Gli2*^*lox/lox*^ (*Atoh1-Gli2* CKO, red) mice. All of the analyses were performed on 3 midline sections per brain. All graphical data are presented as means ± SEM and significance determined using two-tailed T-test. **g**-**h** In situ hybridization of *Cre* RNA on P8 midsagittal cerebellar sections of *Gli2*^*lox/lox*^ (control, **g**) and *Atoh1-Cre/+; Gli2*^*lox/lox*^ (*Atoh1-Gli2* CKO, **h**) mice. Black arrows indicate the loss of *Cre* expression in the partially rescued EGL. **i**-**k** FIHC detection of the indicated proteins and dapi on P8 mid-sagittal cerebellar sections of *Gli2*^*lox/lox*^ (control, **j**) and *Atoh1-Cre/+; Gli2*^*lox/lox*^ (*Atoh1-Gli2* CKO, **i** and **k**) mice. EGL is indicated by the yellow dotted lines and yellow asterisk indicates low level of SOX2 expression in the mutant EGL . Scale bars represent 100 μm

Since the final size of the CB is largely dependent on the expansion of the EGL, we analyzed *Atoh1-Gli2* CKO cerebella at P8 when the EGL is normally thick. Similar to our previous study using irradiation at P1 [25], the EGL was replenished with proliferating cells by P8 in *Atoh1-Gli2* CKO animals. We therefore performed in situ hybridization (ISH) and analyzed the expression of *Gli1*. Although *Gli1* expression was greatly diminished at P0 (Fig. 1h), the EGL of P8 *Atoh1-Gli2* CKOs exhibited *Gli1* expression throughout the anterior EGL, comparable to that observed in control animals and the posterior EGL of mutants (Fig. 4a-b). In addition and unlike at P0, no difference in *Gli1* expression was observed in the PCL at P8 (Fig. 4a-b). Moreover, *Gli1*+ cells expanded in the EGL, as revealed by the proliferation maker Ki67 (Fig. 4c-d). We measured the thickness of the EGL and found no significant difference between control and *Atoh1-Gli2* CKO P8 animals (*n* = 4; 0.044 ± 0.0025 mm vs 0.0399 ± 0.0023 mm) (Fig. 4e). However we observed a significant increase in the size of the outer EGL (oEGL) by seemingly delaying their differentiation compared to controls as revealed by the increase [oEGL/EGL area] ratio (*n* = 4; 68.91 ± 2.44% vs 53.85 ± 0.94%) (Fig. 4f). Analysis of *Cre* expression revealed that only a small subset of cells in the anterior EGL expressed *Cre* in the anterior CB compared to controls (*Atoh1-Cre/+; Gli2*^*flox/+*^ or *Atoh1-Gli2* het) (Fig. 4g-h). Consistent with the presence of wild type (WT) cells in the EGL, GLI2 protein was detected in most cells in the EGL (Fig. 4i-i’). Interestingly, GCPs in the partially rescued anterior EGL expressed a low level of the stem cell marker SOX2 at P8, something that was never observed in control animals (Fig. 4j-k). Interestingly, TUNEL analysis showed a significant increase of cell death in the replenished P8 EGL compared to controls (*n* = 4; 459.9 ± 85.39 vs 89.52 ± 17, 73 particles per mm^2^ of EGL) (Additional file 3: Figure S3a-c) suggesting that some GCPs in the EGL of *Atoh1-Gli2* CKOs that expressed the EGL marker *Atoh1* (as shown by Atoh1-GFP staining, Additional file 3: Figure S3c) turn on *Atoh1-Cre* and die because they lack *Gli2*. Thus, the reduction of GCPs in the EGL of *Atoh1-Gli2* CKOs at P0 stimulates a compensation process that leads to replenishment of the GCPs by WT cells.

### NEPs switch their fate to become GCPs and produce GCs in *Atoh1-Gli2* CKO cerebella

**Fig. 5:**
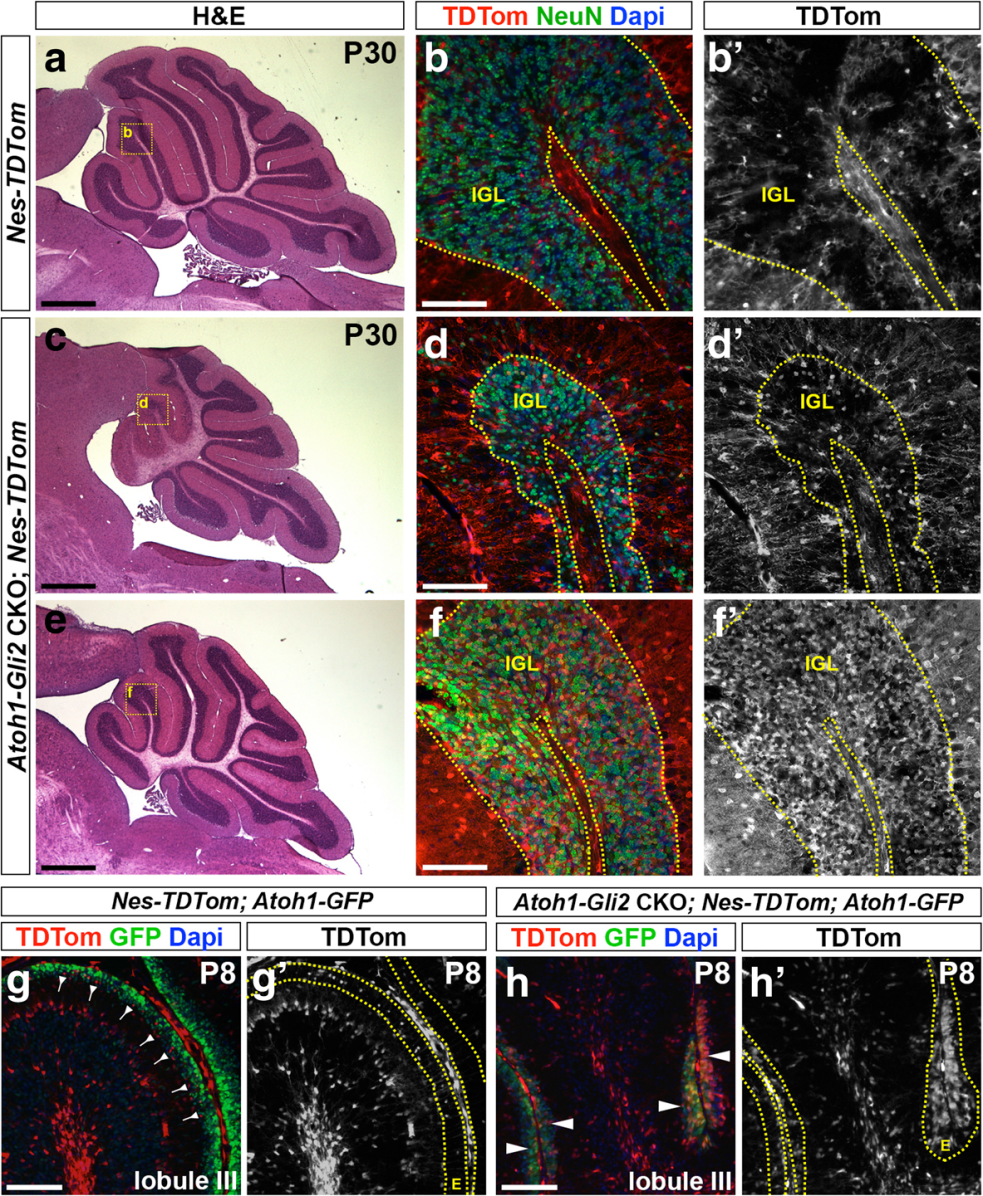
*Nestin*-Expressing Progenitors (NEPs) populate the EGL, express GCP markers and produce granule cells in response to loss of *Gli2* in the EGL. **a**, **c** and **e** H&E staining of sagittal sections of the vermis of P30 *Nes-FlpoER/+; R26*^*FSF-TDTom/+*^ (*Nes-TDTom,* **a**) and *Atoh1-Cre/+; Gli2*^*lox/lox*^*; Nes-FlpoER/+; R26*^*FSF-TDTom/+*^ (*Atoh1-Gli2* CKO*; Nes-TDTom,* **c** and **e**) mice injected with Tm at P0. **b**, **d** and **f** FIHC detection of the indicated proteins and dapi on mid-sagittal cerebellar sections at P30. High power images are shown of the areas indicated by yellow rectangles in (**a**, **c** and **e**). IGL is outlined by yellow dotted lines. **g**-**h** FIHC detection of the indicated proteins and dapi on mid-sagittal cerebellar sections (lobule III) of P8 *Nes-FlpoER/+; R26*^*FSF-TDTom/+*^*; Atoh1-GFP/+* (*Nes-TDTom; Atoh1-GFP,* **g**) and *Atoh1-Cre/+; Gli2*^*lox/lox*^*; Nes-FlpoER/+; R26*^*FSF-TDTom/+/+*^*; Atoh1-GFP/+* (*Atoh1-Gli2* CKO*; Nes-TDTom; Atoh1-GFP,* **h**) mice injected with Tm at P0. The EGL (E) is outlined by yellow dotted lines. Backward arrows indicate TDTom+ and Atoh1-GFP- cells in the inner EGL. White arrowheads indicate TDTom+ and Atoh1-GFP+ cells in the inner EGL. Scale bars represent 1 mm (**a**, **c** and **e**) and 100 μm (**b**, **d**, **f**, **g** and **h**)

Our previous study demonstrated that SOX2+ NEPs can migrate to the EGL after injury and then turn off SOX2 and turn on *Atoh1* [25], and our present results revealed that some cells in the rescued EGL in P8 *Atoh1-Gli2* CKO cerebella express a low level of SOX2. We thus hypothesized that WT NEPs (SOX2+) are able to change their fate to become GCPs and replenish the EGL as part of a compensation mechanism in *Atoh1-Gli2* CKOs. Using a *Nestin-FlpoER* transgene [40] and a Flippase (Flp)-dependent *R26* reporter allele that expresses TDTom, we performed genetic inducible fate mapping (GIFM) of NEPs in the *Atoh1-Gli2* CKO cerebella. In contrast to P30 controls (*Nestin-FlpoER/+*; *R26*^*frt-STOP-frt-TDTom/+*^ or *Nes-TDTom* mice administrated Tm at P0), we observed many TDTom+ cells that also expressed the GC marker NeuN in the IGL of *Atoh1-Gli2* CKO mutants (*n* = 6) (Fig. 5a-f). Interestingly, the vermis of P30 *Atoh1-Gli2* CKOs that recovered well showed an apparently greater contribution of NEP-derived TDTom+ cells in the IGL than those mice that recovered poorly (compare Fig. 5c-d to e-f). Similar results were obtained when analyzing the hemispheres (Additional file 4: Figure S4). However, and consistent with our analysis of CB size, there appeared to be less variability in the percentage of TDTom+ cells observed in the hemispheres compared to the vermis. Analysis of the vermis at P8 showed a significant increase in the number of *Nestin*-derived TdTom+ cells in the EGL compared to controls (*n* = 3; 3049 ± 713.6 vs 308.8 ± 121 TDTom+ cells per mm^2^ of EGL; *p* = 0.0193) (Fig. 5g-h). Furthermore, TDTom+ cells in the P8 EGL already expressed the GCP marker *Atoh1* (as shown by Atoh1-GFP staining) (*n* = 3; 80.92 ± 3.69 vs 1.74 ± 1.11% of TDTom+ Atoh1-GFP+/ total TDTom+ cells in the EGL; *p* < 0.0001) (Fig. 5g-h).

### A subset of proliferating PCL NEP-derived cells migrate to the IGL in *Atoh1-Gli2* CKO cerebella

**Fig. 6:**
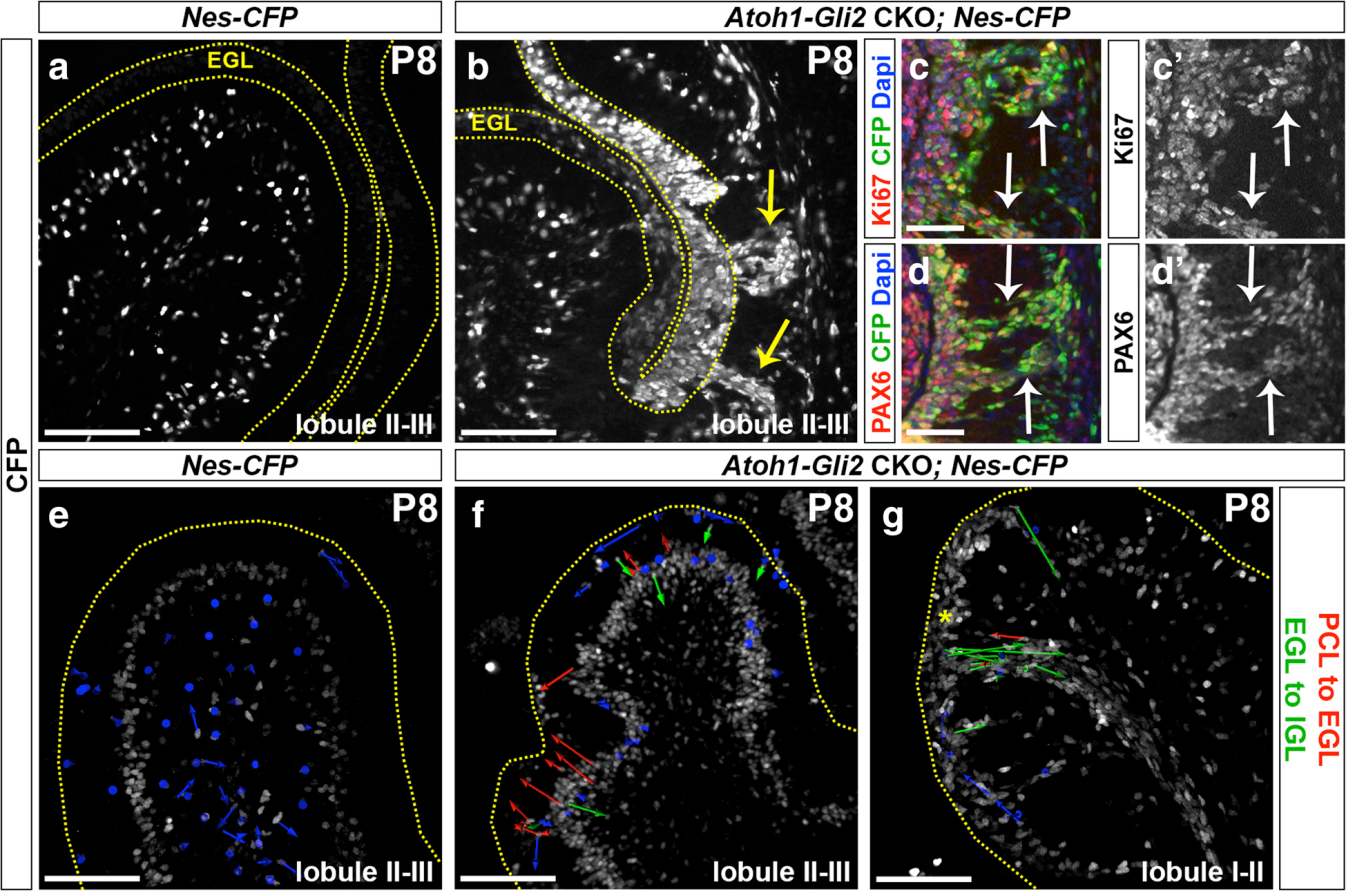
PCL NEPs migrate to the EGL and a subset of proliferating NEP-derived GCP-like cells migrate back into the cerebellar cortex in streams. **a**-**b** FIHC detection of CFP protein on mid-sagittal cerebellar sections (lobule II-III) of P8 *Nes-CFP/+* (*Nes-CFP,* **a**) and *Atoh1-Cre/+; Gli2*^*lox/lox*^*; Nes-CFP/+* (*Atoh1-Gli2* CKO*; Nes-CFP,* **b**) mice. The EGL is indicated by the yellow dotted lines and yellow arrows indicate the streams. **c-d** FIHC detection of the indicated proteins and dapi on high magnifications focusing on CFP+ streams of mid-sagittal cerebellar sections of P8 *Atoh1-Cre/+; Gli2*^*lox/lox*^*; Nes-CFP/+* (*Atoh1-Gli2* CKO*; Nes-CFP*) mice. White arrows indicate co-localization of CFP with the indicated protein. **e**-**g** Detection of native CFP fluorescence on sagittal slice cultures of the vermis (lobule II-III, **e** and **f** and lobule I-II, **g**) of P8 *Nes-CFP/+* (*Nes-CFP,* **e**) and *Atoh1-Cre/+; Gli2*^*lox/lox*^*; Nes-CFP/+* (*Atoh1-Gli2* CKO*; Nes-CFP,* **f** and **g**) mice showing displacement of CFP+ cells during 6 h of imaging. Arrow color code is as indicated (red arrows indicates NEPs moving from PCL to EGL and green arrows indicates NEPs moving from EGL to IGL). The upper edge of the EGL is indicated by a yellow dotted line. Scale bars represent 100 μm (**a**-**b** and **e**-**g**) and 50 μm (**c**-**d**)


We next analyzed the behavior of NEPs using a *Nes-CFP* reporter line [43]. Consistent with our GIFM experiment and unlike control animals (*Nes-CFP*), the EGL of *Atoh1-Gli2* CKOs expressed a high level of CFP (Fig. 6a-b). Surprisingly, streams of CFP+ cells were seen in lobule 3 spanning between the IGL and EGL that were not present in controls (Fig. 6a-b) or in irradiated mice [25]. Interestingly, some cells in the streams expressed the proliferation maker Ki67 as well as the GCP/GC marker PAX6 (Fig. 6c-d) suggesting that a subset of NEP-derived cells were not able to stay in the EGL and thus migrated back to the cerebellar cortex. To test this idea we performed live imaging of P8 *Nes-CFP* cerebellar slice cultures from both control and *Atoh1-Gli2* CKOs (*Atoh-Gli2* CKO; *Nes-CFP*). Strikingly, by tracking the movement of individual cells during ~ 6 h of imaging we observed Nes-CFP+ cells actively migrating from the PCL to the EGL in slices from *Atoh-Gli2* CKO cerebella but not control mice at P8 (Fig. 6e-f and Additional file 5: Video S1 and Additional file 6: Video S2). The CFP+ layer of cells also appeared thicker in the mutants, indicating the NEPs expanded in number. Interestingly, in the areas containing streams of CFP+ cells the majority of cells that were tracked moved in the opposite direction from the EGL to the IGL (Fig. 6g and Additional file 7: Video S3). Our live imaging experiments thus provide evidence that NEPs located in the PCL expand and then migrate to replenish the EGL in response to GCP-specific loss of *Gli2*. Furthermore, a subset of NEP-derived cells is not able to integrate into the EGL and migrate back down to towards the cerebellar cortex.

**Additional file 5: Video S1.** P8 WT cerebellum shows no obvious movement of NEPs towards the EGL. Detection of native CFP fluorescence on sagittal slices of the vermis (lobule 2/3) of P8 *Nes-CFP/+* mice showing displacement of CFP+ cells. Image stacks were acquired every 5 min for 4 h. (MOV 3481 kb)


### *Gli2* CKO in NEPs inhibits the recovery of the EGL in *Atoh1*-*Gli2* CKOs

**Fig. 7:**
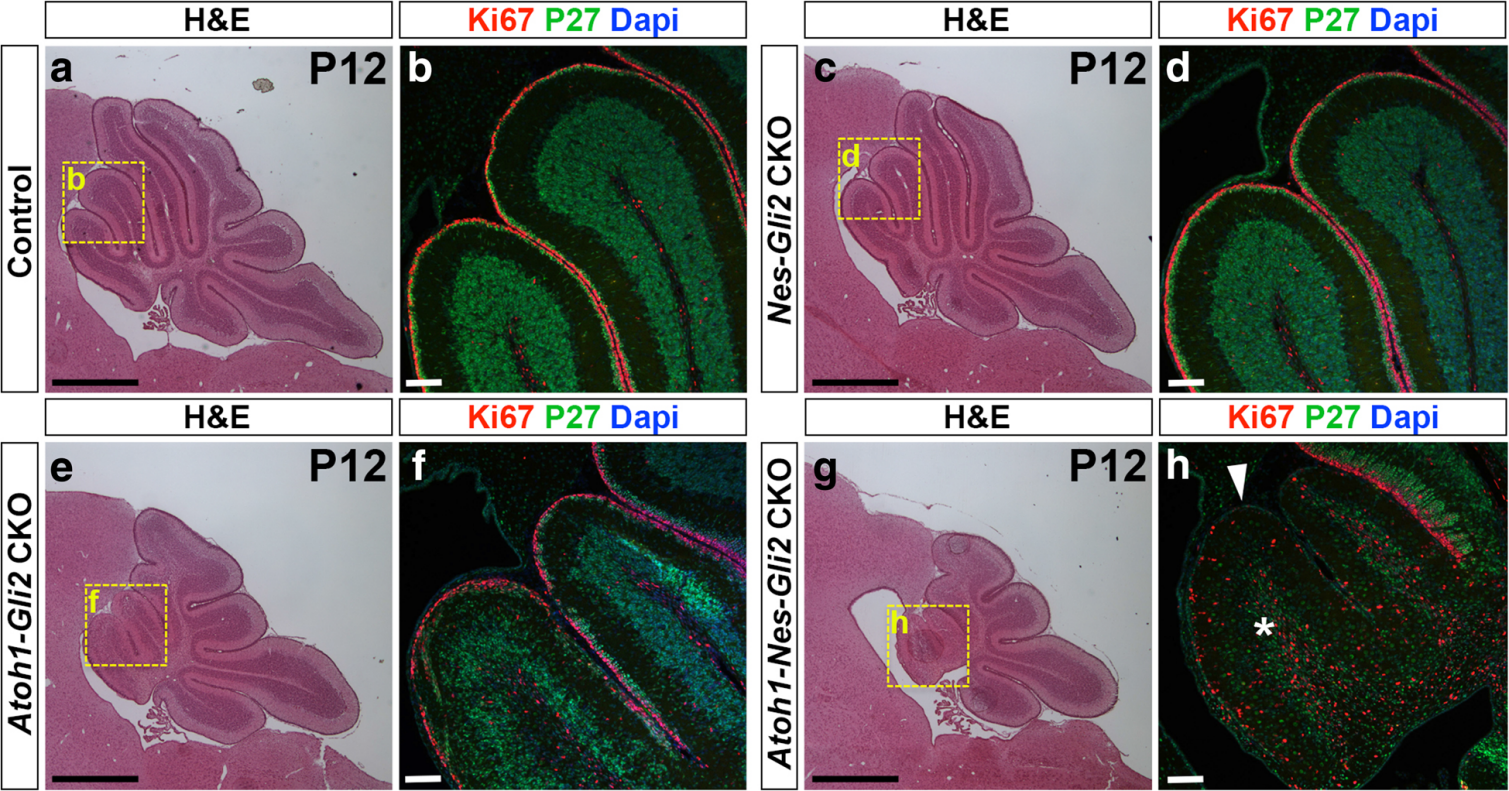
Inactivation of *Gli2* in both *Nestin-* and *Atoh1*-expressing cells inhibits the recovery of the CB compared to in mice lacking *Gli2* only in the EGL. **a**, **c**, **e** and **g** H&E of sagittal sections of the cerebellar vermis of P12 *Gli2*^*flox/flox*^ (Control, **a**), *Nes-FlpoER/+; R26*^*MASTR/+*^*; Gli2*^*flox/flox*^ (*Nes-Gli2* CKO, **c**), *Atoh1-Cre/+; Gli2*^*flox/flox*^ (*Atoh1-Gli2* CKO, **e**), and *Atoh1-Cre/+; Nes-FlpoER/+; R26*^*MASTR/+*^*; Gli2*^*flox/flox*^ (*Atoh1-Nes-Gli2* CKO, **g**) mice injected with Tm at P0. Note that inactivation of *Gli2* only in *Nestin*-expressing cells has no major effect of CB development at P12. However, inactivation of *Gli2* in *Nestin*-expressing cells inhibits the compensation mechanism when *Gli2* is removed in GCPs (**g** compared to **e**). **b**, **d**, **f** and **h** High magnifications (as shown by yellow squares in (**a**, **c**, **e** and **g**) of anterior vermis of P12 *Gli2*^*flox/flox*^ (**b**), *Nes-FlpoER/+; R26*^*MASTR/+*^*; Gli2*^*flox/flox*^ (*Nes-Gli2* CKO, **d**), *Atoh1-Cre/+; Gli2*^*flox/flox*^ (*Atoh1-Gli2* CKO, **f**), and *Atoh1-Cre/+; Nes-FlpoER/+; R26*^*MASTR/+*^*; Gli2*^*flox/flox*^ (*Atoh1-Nes-Gli2* CKO, **h**) cerebella stained with the indicated proteins and dapi. White arrowhead and white asterisk indicate the loss of EGL and IGL respectively in *Atoh1-Nes-Gli2* CKO. Scale bars represent 1 mm (**a**, **c**, **e** and **g**) and 100 μm (**b**, **d**, **f** and **h**)

**Fig. 8:**
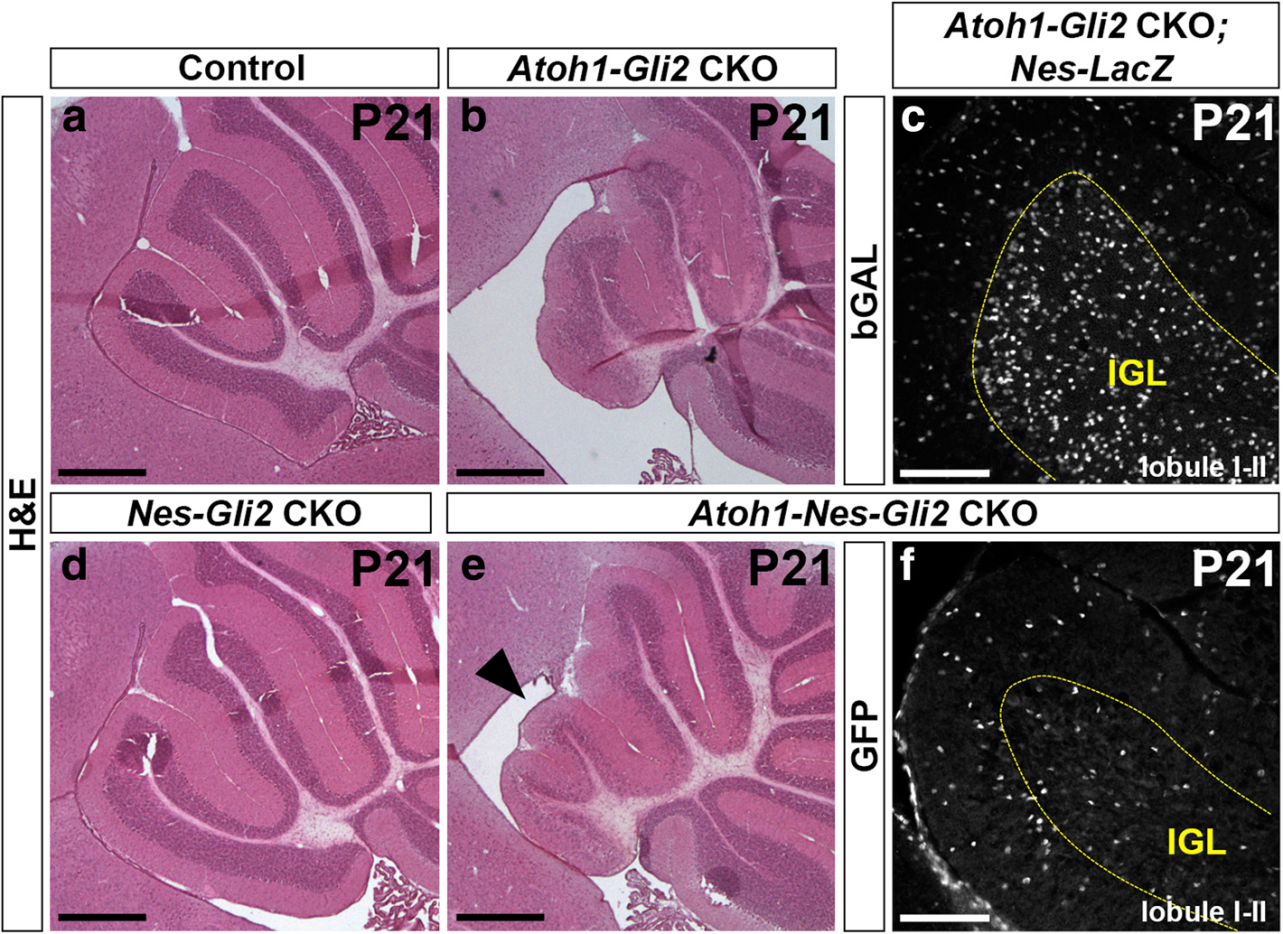
NEP-derived cells failed to populate the IGL at P21. **a**, **b**, **d** and **e** H&E staining of sagittal sections of anterior cerebellar vermis of P21 *Gli2*^*flox/flox*^ (Control, **a**), *Atoh1-Cre/+; Gli2*^*flox/flox*^ (*Atoh1-Gli2* CKO, **b**), *Nes-FlpoER/+; R26*^*MASTR/+*^*; Gli2*^*flox/flox*^ (*Nes-Gli2* CKO, **d**) and *Atoh1-Cre/+; Nes-FlpoER/+; R26*^*MASTR/+*^*; Gli2*^*flox/flox*^ (*Atoh1-Nes-Gli2* CKO, **e**) mice injected with Tm at P0. Note that inactivation of *Gli2* in *Nestin*-expressing cells inhibits the compensation mechanism in the anterior vermis (black arrowhead in **e**). **c** and **f** FIHC detection of the indicated proteins on mid-sagittal cerebellar sections (lobule I-II) of P21 *Atoh1-Cre/+; Gli2*^*flox/flox*^; *Nes-FlpoER/+; R26*^*FSF-LacZ/+*^ (*Atoh1-Gli2* CKO*; Nes-LacZ*, **c**) and *Atoh1-Cre/+; Nes-FlpoER/+; R26*^*MASTR/+*^*; Gli2*^*flox/flox*^ (*Atoh1-Nes-Gli2* CKO, **f**) mice injected with Tm at P0. IGL is indicated by the yellow doted line. Scale bars represent 500 μm (**a**, **b**, **d** and **e**) and 100 μm (**c** and **f**)

Since we have shown previously that SHH signaling (*Smo*) is necessary in NEPs for CB recovery following irradiation, we tested whether *Gli2* plays a role in this process. We generated littermates of 4 different genotypes that were administered Tm at P0, and analyzed each genotype (*n* = 3) at P8, P12 and P21: *Gli2*^*flox/flox*^ WT (control) mice, *Nestin-FlpoER/+; R26*^*MASTR/+*^; *Gli2*^*flox/flox*^ single (*Nes-Gli2* CKOs mutants lacking *Gli2* in NEPs), *Atoh1-Cre/+; Gli2*^*flox/flox*^ single (*Atoh1-Gli2* CKOs lacking *Gli2* in anterior GCPs) and *Nestin-FlpoER/+; R26*^*MASTR/+*^; *Atoh1-Cre/+; Gli2*^*flox/flox*^ double (*Atoh1-Nes-Gli2* CKOs lacking *Gli2* in NEPs and GCPs) mutants. We did not observe any obvious phenotype in the *Nes-Gli2* CKOs at all stages compared to controls (compare Fig. 7c-d to a-b, Additional file 8: Figure S5c-d to a-b and Fig. 8d to a). However, H&E staining revealed that the anterior CB appeared reduced in the double mutants (*Atoh1-Nes-Gli2* CKOs) compared to *Atoh1-Gli2* CKOs at both P8 and P12 (compare Fig. 7e to g and Additional file 8: Figure S5e to g). Analysis of proliferating GCPs in the outer EGL (Ki67+) and post mitotic GCs in the inner EGL and IGL (P27+) at both stages showed that the anterior EGL appeared more depleted in *Atoh1-Nes-Gli2* CKO compared to *Atoh1-Gli2* CKO cerebella and that *Atoh1-Nes-Gli2* CKO animals failed to form a proper P27+ inner EGL and IGL (compare Fig. 7f to h and Additional file 8: Figure S5f to h). Analysis of the fate of GFP+ *Nestin*-expressing cells in P21 *Atoh1-Nes-Gli2* CKOs using a Flp-mediated *R26* reporter allele that expresses nuclear βGalactosidase (Bgal) (*R26*^*frt-STOP-frt-lacZ/+*^) revealed that unlike *Atoh1-Gli2* CKOs in which *Nestin*-derived GCs populated the IGL (Figs. 5a-f and 8c), the *Gli2* mutant *Nestin*-derived cells populated the IGL poorly (Fig. 8f). The few GFP+ cells in the IGL of the double mutants were likely interneurons or astrocytes.

These results demonstrate that SHH-signaling through GLI2 plays a crucial role during NEP-mediated cerebellar recovery from loss of GCPs.

## Discussion

In this study we developed a conditional mutant strategy to delete *Gli2* (the gene encoding the major effector of SHH signaling) in most GCPs in the anterior cerebellum using an *Atoh1-Cre* transgene used in many studies (e.g. [41, 45–52]). Although we show using a mosaic analysis that SHH-GLI2 signaling is crucial for generating the correct pool of GCs by promoting GCP viability and proliferation, deletion of *Gli2* in the EGL using this transgene is not sufficient to induce a major hypoplasia of the adult cerebellum in most mutants. We discovered that although the GCP pool is greatly diminished in *Atoh1-Gli2* neonates, it is subsequently replenished by a cellular mechanism that includes adaptive reprogramming of WT NEPs to become GCPs. Importantly, since the transgene does not turn on in many of the newly generated GCPs, the EGL recovers and generates GCs. Rare GCPs that never express *Cre* could also contribute to replenishment of the GCPs if they can undergo more rounds of cell division than normal.

A question raised by this and previous studies is what signals induce the NEPs that reside in the PCL to change their fate and become GCPs. It was previously shown that when irradiation is used to kill most GCPs at P2–3, NEPs contribute to replenishment of the EGL [25]. It is therefore possible that in *Atoh1-Gli2* mutants a signal associated with the cell death we observed triggers NEPs to change their fate and generate GCPs. An alternative mechanism is that the depletion of the EGL results in a change in Purkinje cell signaling, possibly because of a reduction in excitatory input since less granule cells are generated. In turn, SHH might preferentially accumulate in the cell bodies of Purkinje cells, which lie close to the NEPs, thus increasing HH signaling to NEPs [25]. Consistent with a role for GCs in regulating NEP behaviors, the *Atoh1-Cre* transgene is first expressed in GCPs at E13.5 [45], but the replenishment of the EGL in *Atoh1-Gli2* CKOs only occurs several days after the EGL is depleted and when the IGL normally first becomes apparent (P3-P4). Thus, a possible involvement of NEPs in a compensation processes should be considered for conditional mutants that alter not only GCP proliferation and survival, but also genes involved in differentiation of GCs.

It might be expected that *Atoh1-Smo* CKOs would have a similar phenotype to *Atoh1-Gli2* CKOs, given that both genes are required for SHH signaling. However, *Atoh1-CreER/+; Smo*^*loxP/Δ*^ mice in which one allele of the *Smoothened* gene is deleted in the germline and deletion of the floxed allele is dependent on tamoxifen (Tm) administration have a severe cerebellum hypoplasia [13]. A possible explanation for the phenotype in such mutants is that reprogramming of NEPs in *Smo* heterozygous mutants is partially compromised since HH signaling is crucial for the expansion of PCL NEPs and their migration to the EGL [25]. In addition, since Tm diminishes cerebellum recovery after EGL depletion [25], the combination of a lower level of SMO protein in NEPs and administration of Tm to *Atoh1-CreER/+; Smo*^*loxP/Δ*^ mice might blunt the response of NEPs to *Smo*-dependent depletion of the EGL leading to severe hypoplasia.

We observed a large variability in the vermal hypoplasia of *Atoh1-Gli2* CKO adults, suggesting that only some mutant cerebella can efficiently recover. The variability in recovery is likely because the degree to which the *Atoh1*-*Cre* transgene is turned on in newly formed WT GCPs varies between mice. Since loss of *Gli2* leads to cell death, the TUNEL staining observed in the EGL of P8 *Atoh1-Gli2* CKO cerebella is consistent with *Gli2* being deleted after P6 in some new wild type GCPs that are either derived from NEPs or rare GCPs that had not previously expressed *Atoh1-Cre*. In addition, our experiments indicate that some NEPs that enter the EGL and turn on PAX6 migrate back towards the IGL before becoming postmitotic and turning off Nes-CFP. We hypothesize that some NEP-derived GCPs that undergo deletion of *Gli2* after entering the EGL survive but are unable to fully reprogram into GCPs. An interesting gene that might not be properly turned on is *CxcR4*, since SDF1 expressed by meninges signals through CXCR4 to maintain GCPs in the outer EGL and to enhance SHH-dependent proliferation [53]. Furthermore, SHH-GLI1 signaling induces the transcription of *Cxcr4* and *Cxcr7* [54]. We propose that in *Atoh1-Gli2* CKOs a subset of PCL-derived NEPs express CRE after entering the EGL, and the subsequent loss of GLI2 protein reduces CXCR4, leading to migration of proliferating GCP-like cells back into the cerebellar cortex. Thus, the variable extent of growth of the cerebellum in *Atoh1-Gli2* CKOs likely results from the amount of *Atoh1-Cre* induced after P4 in GCPs, and the resulting balance of cell death and premature migration from the EGL.

The cerebellum is broadly divided along the medio-lateral axis into a central vermis and two lateral hemispheres [19]. Although recovery from depletion of the EGL at P0 occurs in both regions of *Atoh1-Gli2* CKOs, the recovery is more robust in the hemispheres. Curiously, the hemispheres of *Atoh1-Gli2* CKOs exhibit extra folds at P8 (arrow in Fig. 3g), suggesting a differential recovery response along the medio-lateral axis. The vermis and hemispheres are molecularly and functionally distinct [19, 55], and hemispheric GCPs have a higher sensitivity to high-level SHH-signaling than those in the vermis [55]. We propose that hemispheric NEP-derived GCPs in the EGL maintain a higher level of SHH signaling and therefore expand more rapidly and efficiently than those in the vermis.

## Conclusion

In this study, we show that the ability of NEPs to compensate for postnatal cerebellar damage must be considered in the interpretation of any mutant phenotype where genes involved in EGL cell proliferation/differentiation and survival have been disrupted. This is particularly the case if the *Atoh1-Cre* transgene utilized in this study [41] is employed to generate conditional mutants. Compensation for a loss of GCPs is most likely to occur for genes that are required after birth, once NEPs are present. Finally, our findings raise the question of whether similar recovery phenomena occur in other regions of the brain, and depending on the transgene used could complicate interpretation of mutant phenotypes.

## Additional files

Additional file 1: **Figure S1.** Similar to in the vermis, SHH-Gli2 maintains GCP in an undifferentiated state and promotes their survival in the hemispheres. (a) Graphs of the number of GFP+ cells in the outer (o) EGL at P4 (*n* = 3) and P8 (*n* = 3) in both hemispheres and vermis of *R26*^*MASTR/+*^; *Atoh1-FlpoER/+; Gli2*^*lox/+*^ (*Atoh1-M-Gli2* het, black) and *R26*^*MASTR/+*^; *Atoh1-FlpoER/+; Gli2*^*lox/lox*^ (*Atoh1-M-Gli2* CKO, red) mice treated with Tm at P2. (b-d) Graphs of the proportion of CFP+ cells in the outer (o) EGL at P8 (*n* = 3) (b), the proliferation index at P8 (% [GFP+ EdU+] cells of all [GFP+] cells in the oEGL) (*n* = 3) (c) and the number of TUNEL+ particles per section at P4 (*n* = 3) (d) in the hemispheres of *R26*^*MASTR/+*^; *Atoh1-FlpoER/+; Gli2*^*lox/+*^ (*Atoh1-M-Gli2* het, black) and *R26*^*MASTR/+*^; *Atoh1-FlpoER/+; Gli2*^*lox/lox*^ (*Atoh1-M-Gli2* CKO, red) mice treated with Tm at P2. All of the analyses were performed on 3 sections per region and per brain. All graphical data are presented as means ± SEM and significance determined using two-tailed. (JPG 238 kb)

Additional file 2: **Figure S2.** GLI2 protein is lost in the P0 *Atoh1-Gli2* CKO EGL. (a and d) In situ hybridization of *Cre* mRNA on P0 mid-sagittal cerebellar sections of *Gli2*^*lox/lox*^ (control, a) and *Atoh1-Cre/+; Gli2*^*lox/lox*^ (*Atoh1-Gli2* CKO, d) mice. Black arrows indicate the lack of *Cre* expression in the most posterior part of the CB. (b-c and e-f) FIHC detection of GLI2 protein and dapi in the indicated regions (as shown by black squares in a and d) in P0 *Gli2*^*lox/lox*^ (control, b-c) and *Atoh1-Cre/+; Gli2*^*lox/lox*^ (*Atoh1-Gli2* CKO, e-f) CB. Yellow arrowhead in e and white arrow in F indicate respectively the absence and presence of GLI2 protein in the EGL. Scale bars represent 1 mm (a and d) and 100 μm (b-c and e-f). (JPG 1811 kb)

Additional file 3: **Figure S3.** Rescued EGL still exhibits an increase in cell death. (a-b) TUNEL and dapi detection on mid-sagittal sections of P8 *Gli2*^*lox/lox*^ (Control) and *Atoh1-Cre/+; Gli2*^*lox/lox*^ (*Atoh1-Gli2* CKO) CB. White arrows indicate the presence of the EGL (b). (c) Graphs of the number of TUNEL+ particles per mm^2^ of EGL (*n* = 4) in the vermis (lobule I to V) of P8 *Gli2*^*lox/lox*^ (Control, black) and *Atoh1-Cre/+; Gli2*^*lox/lox*^ (*Atoh1-Gli2* CKO, red) CB. All of the analyses were performed on 3 sections per region and per brain. All graphical data are presented as means ± SEM and significance determined using two-tailed test. (d) Detection of native GFP fluorescence and in situ hybridization of *Cre* mRNA on a mid-sagittal section (lobule II-III) of a P8 *Atoh1-Cre/+; Gli2*^*lox/lox*^*; Atoh1-GFP/+* (*Atoh1-Gli2* CKO*; Atoh1-GFP*) mouse. EGL is indicated by the yellow doted line and black arrowheads indicate ATOH1-GFP+/ *Cre +* cells*.* Scale bars represent 100 μm (a and b) and 10 μm (d). (JPG 864 kb)

Additional file 4: **Figure S4.** *Nestin*-Expressing Progenitors (NEPs) differentiate into granule neurons in response to loss of *Gli2* in the hemispheres. (a and c) H&E staining of hemispheric sagittal sections of P30 *Nes-FlpoER/+; R26*^*FSF-TDTom/+*^ (*Nes-TDTom,* a) and *Atoh1-Cre/+; Gli2*^*lox/lox*^*; Nes-FlpoER/+; R26*^*FSF-TDTom/+*^ (*Atoh1-Gli2* CKO*; Nes-TDTom,* c) mice injected with Tm at P0. (b and d) FIHC detection of the indicated proteins and dapi on hemispheric sagittal cerebellar sections at P30. High power images are shown of the areas indicated by yellow rectangles in (a and c). IGL is indicated by the yellow doted line. Scale bars represent 1 mm (a and c) and 100 μm (b and d). (JPG 1479 kb)

Additional file 6: **Video S2.** PCL NEPs migrate toward the EGL in *Atoh1-Gli2* CKO CB at P8. Detection of native CFP fluorescence on sagittal slices of the vermis (lobule 2/3) of P8 *Atoh1-Cre/+; Gli2*^*flox/flox*^*; Nes-CFP/+* (*Atoh1-Gli2* CKO*; Nes-CFP*) mice showing displacement of CFP+ cells. Image stacks were acquired every 5?min for 4?h. (MOV 1740 kb)

Additional file 7: **Video S3.** A subset of NEP-derived cells migrate from the EGL towards the IGL in *Atoh1-Gli2* CKO CB at P8. Detection of native CFP fluorescence on sagittal slices of the vermis (lobule 1/2) of P8 *Atoh1-Cre/+; Gli2*^*flox/flox*^*; Nes-CFP/+* (*Atoh1-Gli2* CKO*; Nes-CFP*) mice showing displacement of CFP+ cells. Image stacks were acquired every 5?min for 4?h. (MOV 6450 kb)

Additional file 8: **Figure S5.** Inactivation of *Gli2* in both *Nestin* and *Atoh1* expressing cells inhibits the recovery of the CB. (a, c, e and g) H&E staining of sagittal sections of the cerebellar vermis of P8 *Gli2*^*flox/flox*^ (Control, a), *Nes-FlpoER/+; R26*^*MASTR/+*^*; Gli2*^*flox/flox*^ (*Nes-Gli2* CKO, c), *Atoh1-Cre/+; Gli2*^*flox/flox*^ (*Atoh1-Gli2* CKO, e), and *Atoh1-Cre/+; Nes-FlpoER/+; R26*^*MASTR/+*^*; Gli2*^*flox/flox*^ (*Atoh1-Nes-Gli2* CKO, g) mice injected with Tm at P0. Note that inactivation of *Gli2* only in *Nestin*-expressing cells has no major effect at P8. However, inactivation of *Gli2* in *Nestin*-expressing cells inhibits the compensation mechanism (g compared to e). (b, d, f and h) Close-up (as shown by yellow squares in a, c, e and g) of anterior vermis of P8 *Gli2*^*flox/flox*^ (b), *Nes-FlpoER/+; R26*^*MASTR/+*^*; Gli2*^*flox/flox*^ (*Nes-Gli2* CKO, d), *Atoh1-Cre/+; Gli2*^*flox/flox*^ (*Atoh1-Gli2* CKO, f), and *Atoh1-Cre/+; Nes-FlpoER/+; R26*^*MASTR/+*^*; Gli2*^*flox/flox*^ (*Atoh1-Nes-Gli2* CKO, h) cerebella stained with the indicated proteins and dapi. White arrowhead and white asterisk indicate the loss of EGL and IGL respectively in the *Atoh1-Nes-Gli2* CKO. Scale bars represent 1 mm (a, c, e and g) and 100 μm (b, d, f and h). (JPG 2032 kb)

## Abbreviations

A: Activator
CB: Cerebellum
Ci: Cubitus interruptus
CKO: Conditional knockout
*Dhh*: Desert hedgehog
EGL: External Granule Layer
GCP: Granule Cell Precursor
GFP: Green Fluorescent Protein
GIFM: Genetic Inducible Fate Mapping
het: Heterozygous
HH: Hedgehog
IGL: Internal granule cell layer
IHC: immunohistochemistry
*Ihh*: Indian hedgehog
ISH: In situ hybridization
MASTR: Mosaic mutant analysis with spatial and temporal control of recombination
ML: Molecular layer
NEP: Nestin-expressing progenitor
P: Postnatal day
PC: Purkinje cell
PCL: Purkinje cell layer
PTCH1: Patched1
R: Repressor
SHH: Sonic Hedgehog
SMO: Smoothened
TDTom: Tandem Dimeric derivative of DsRed
Tm: Tamoxifen
TUNEL: Terminal deoxynucleotidyl transferase dUTP nick end labeling
VZ: ventricular zone
WT: Wild type
