# Supplementary figures and images for: Genetic deletion of genes in the cerebellar rhombic lip lineage can stimulate compensation through adaptive reprogramming of ventricular zone-derived progenitors
Source: Neural Dev. 2019 Feb 14;14:4. doi: 10.1186/s13064-019-0128-y (PMC6375182; doi:10.1186/s13064-019-0128-y)

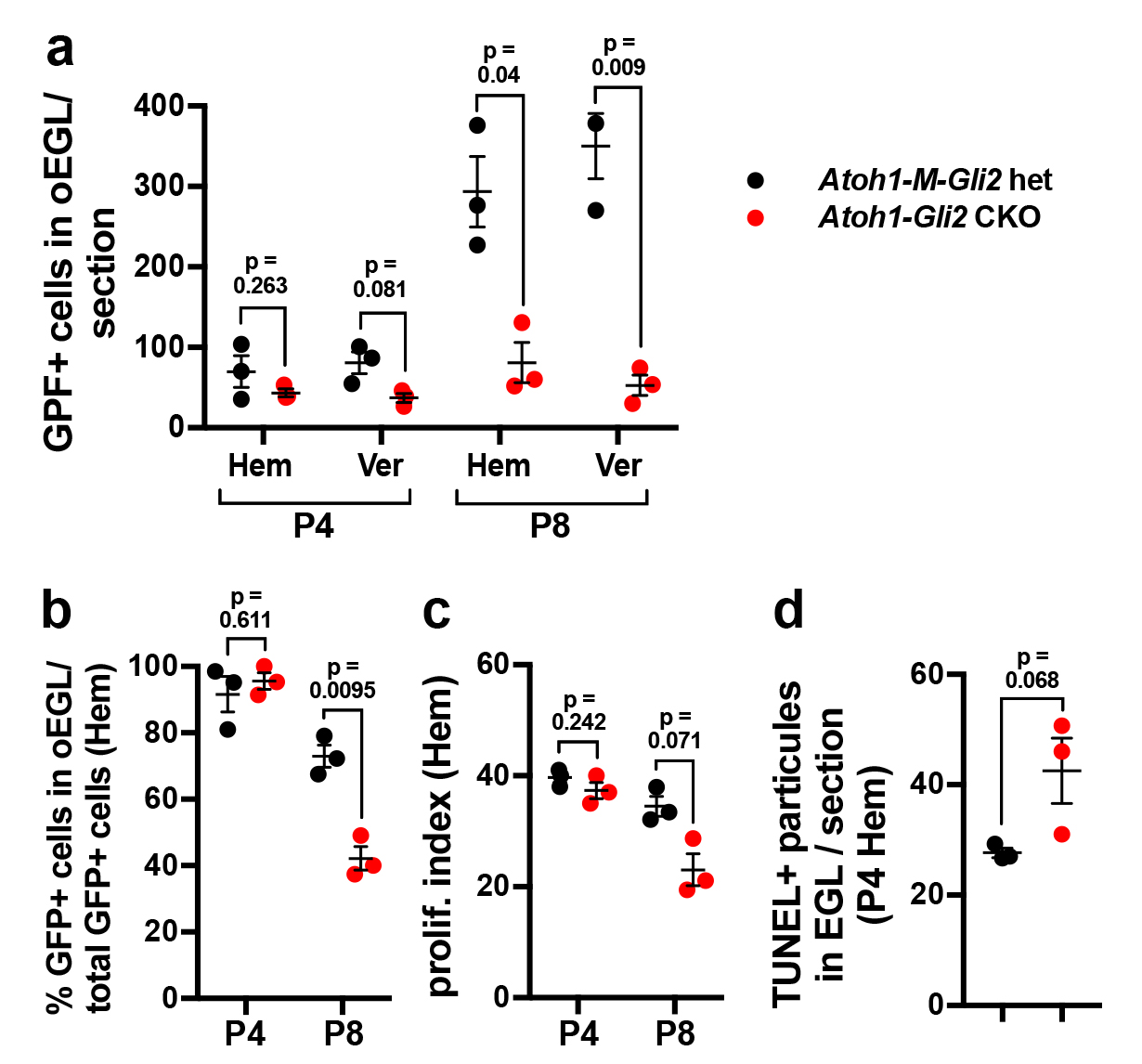

Supplement: Supplementary file 2 — Figure S1. Similar to in the vermis, SHH-Gli2 maintains GCP in an undifferentiated state and promotes their survival in the hemispheres. (a) Graphs of the number of GFP+ cells in the outer (o) EGL at P4 (n = 3) and P8 (n = 3) in both hemispheres and vermis of R26MASTR/+; Atoh1-FlpoER/+; Gli2lox/+ (Atoh1-M-Gli2 het, black) and R26MASTR/+; Atoh1-FlpoER/+; Gli2lox/lox (Atoh1-M-Gli2 CKO, red) mice treated with Tm at P2. (b-d) Graphs of the proportion of CFP+ cells in the outer (o) EGL at P8 (n = 3) (b), the proliferation index at P8 (% [GFP+ EdU+] cells of all [GFP+] cells in the oEGL) (n = 3) (c) and the number of TUNEL+ particles per section at P4 (n = 3) (d) in the hemispheres of R26MASTR/+; Atoh1-FlpoER/+; Gli2lox/+ (Atoh1-M-Gli2 het, black) and R26MASTR/+; Atoh1-FlpoER/+; Gli2lox/lox (Atoh1-M-Gli2 CKO, red) mice treated with Tm at P2. All of the analyses were performed on 3 sections per region and per brain. All graphical data are presented as means ± SEM and significance determined using two-tailed. (JPG 238 kb) [file 13064_2019_128_MOESM1_ESM.jpg]

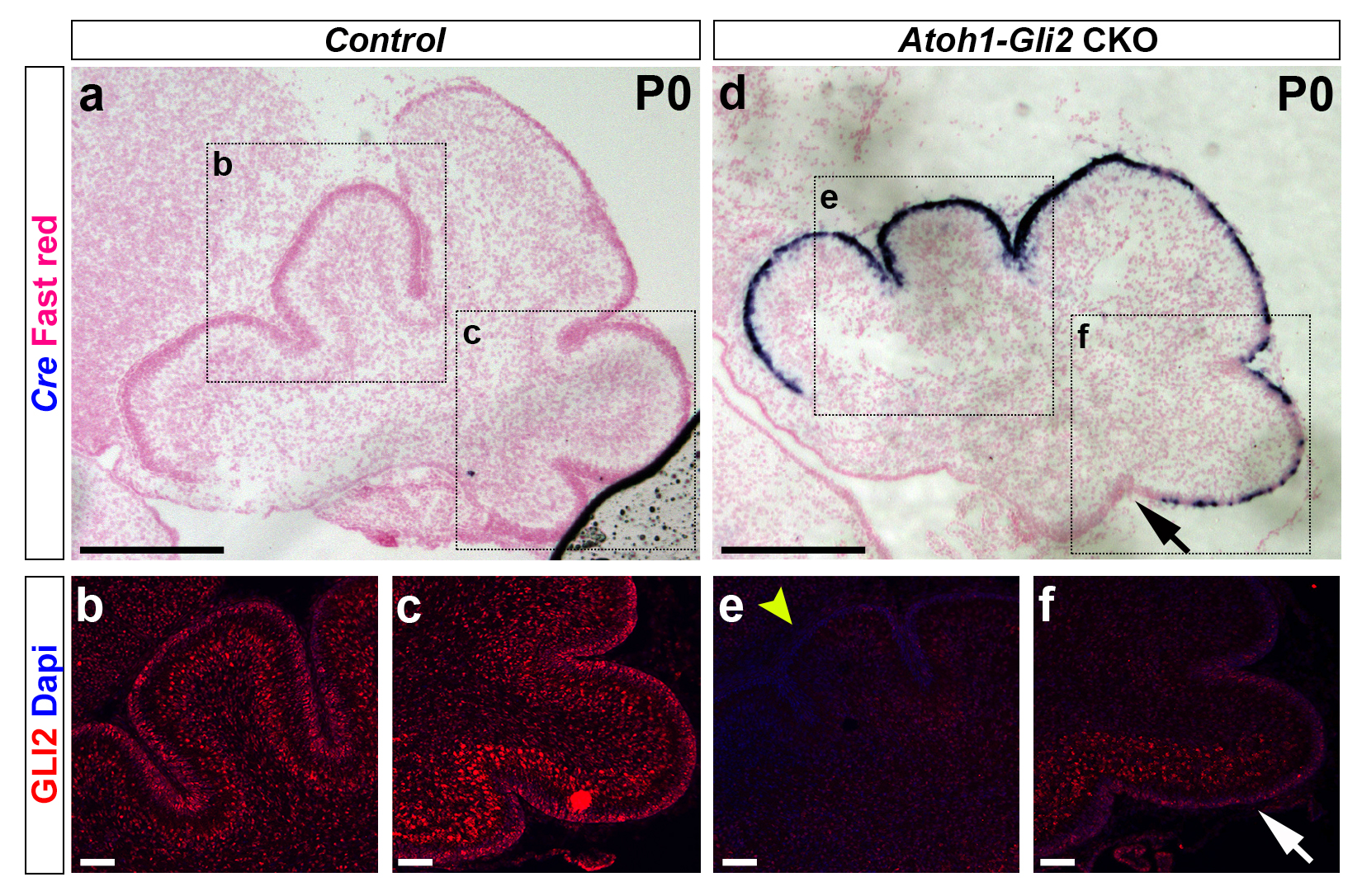

Supplement: Supplementary file 3 — Figure S2. GLI2 protein is lost in the P0 Atoh1-Gli2 CKO EGL. (a and d) In situ hybridization of Cre mRNA on P0 mid-sagittal cerebellar sections of Gli2lox/lox (control, a) and Atoh1-Cre/+; Gli2lox/lox (Atoh1-Gli2 CKO, d) mice. Black arrows indicate the lack of Cre expression in the most posterior part of the CB. (b-c and e-f) FIHC detection of GLI2 protein and dapi in the indicated regions (as shown by black squares in a and d) in P0 Gli2lox/lox (control, b-c) and Atoh1-Cre/+; Gli2lox/lox (Atoh1-Gli2 CKO, e-f) CB. Yellow arrowhead in e and white arrow in F indicate respectively the absence and presence of GLI2 protein in the EGL. Scale bars represent 1 mm (a and d) and 100 μm (b-c and e-f). (JPG 1811 kb) [file 13064_2019_128_MOESM2_ESM.jpg]

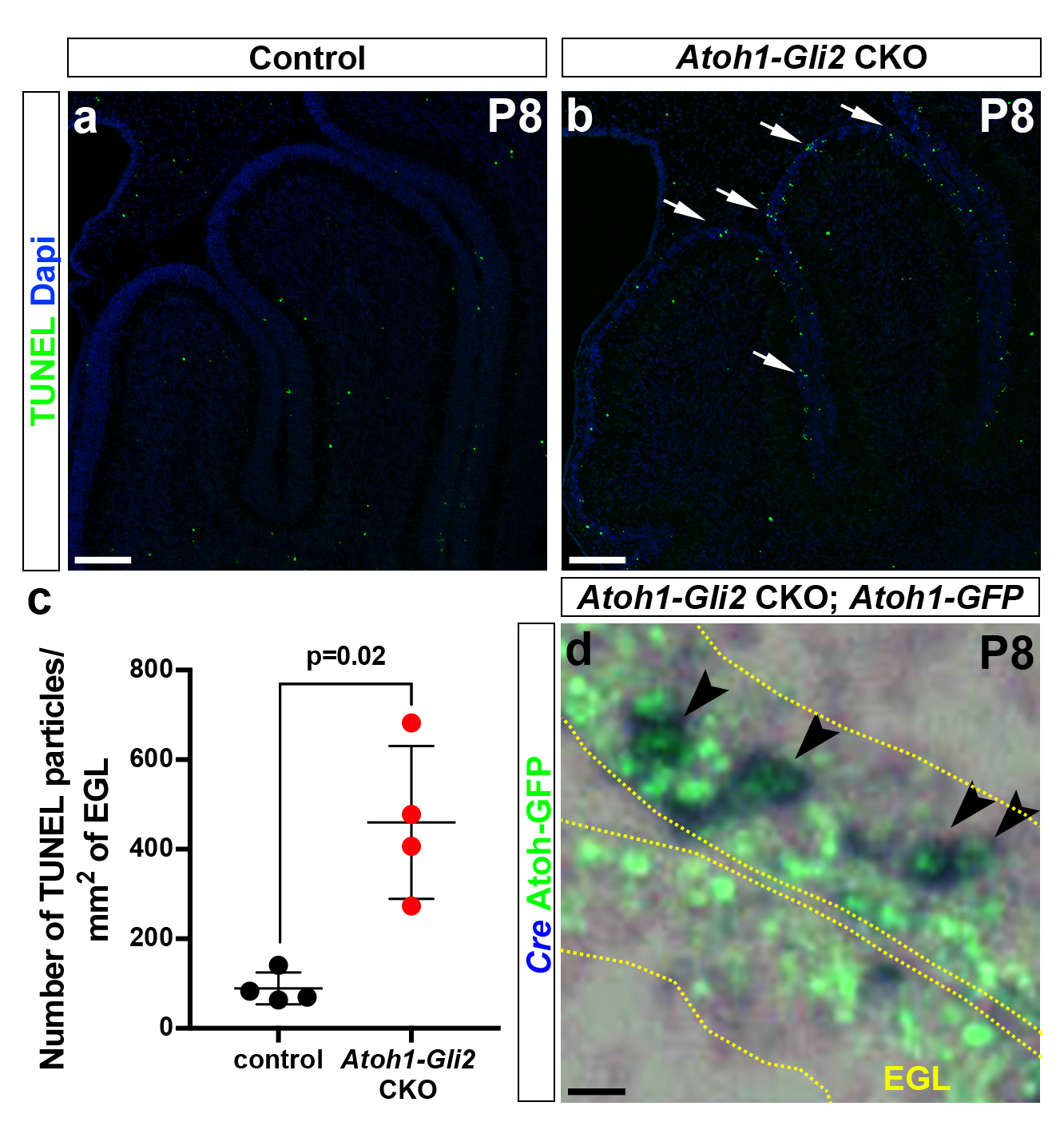

Supplement: Supplementary file 4 — Figure S3. Rescued EGL still exhibits an increase in cell death. (a-b) TUNEL and dapi detection on mid-sagittal sections of P8 Gli2lox/lox (Control) and Atoh1-Cre/+; Gli2lox/lox (Atoh1-Gli2 CKO) CB. White arrows indicate the presence of the EGL (b). (c) Graphs of the number of TUNEL+ particles per mm2 of EGL (n = 4) in the vermis (lobule I to V) of P8 Gli2lox/lox (Control, black) and Atoh1-Cre/+; Gli2lox/lox (Atoh1-Gli2 CKO, red) CB. All of the analyses were performed on 3 sections per region and per brain. All graphical data are presented as means ± SEM and significance determined using two-tailed test. (d) Detection of native GFP fluorescence and in situ hybridization of Cre mRNA on a mid-sagittal section (lobule II-III) of a P8 Atoh1-Cre/+; Gli2lox/lox; Atoh1-GFP/+ (Atoh1-Gli2 CKO; Atoh1-GFP) mouse. EGL is indicated by the yellow doted line and black arrowheads indicate ATOH1-GFP+/ Cre + cells. Scale bars represent 100 μm (a and b) and 10 μm (d). (JPG 864 kb) [file 13064_2019_128_MOESM3_ESM.jpg]

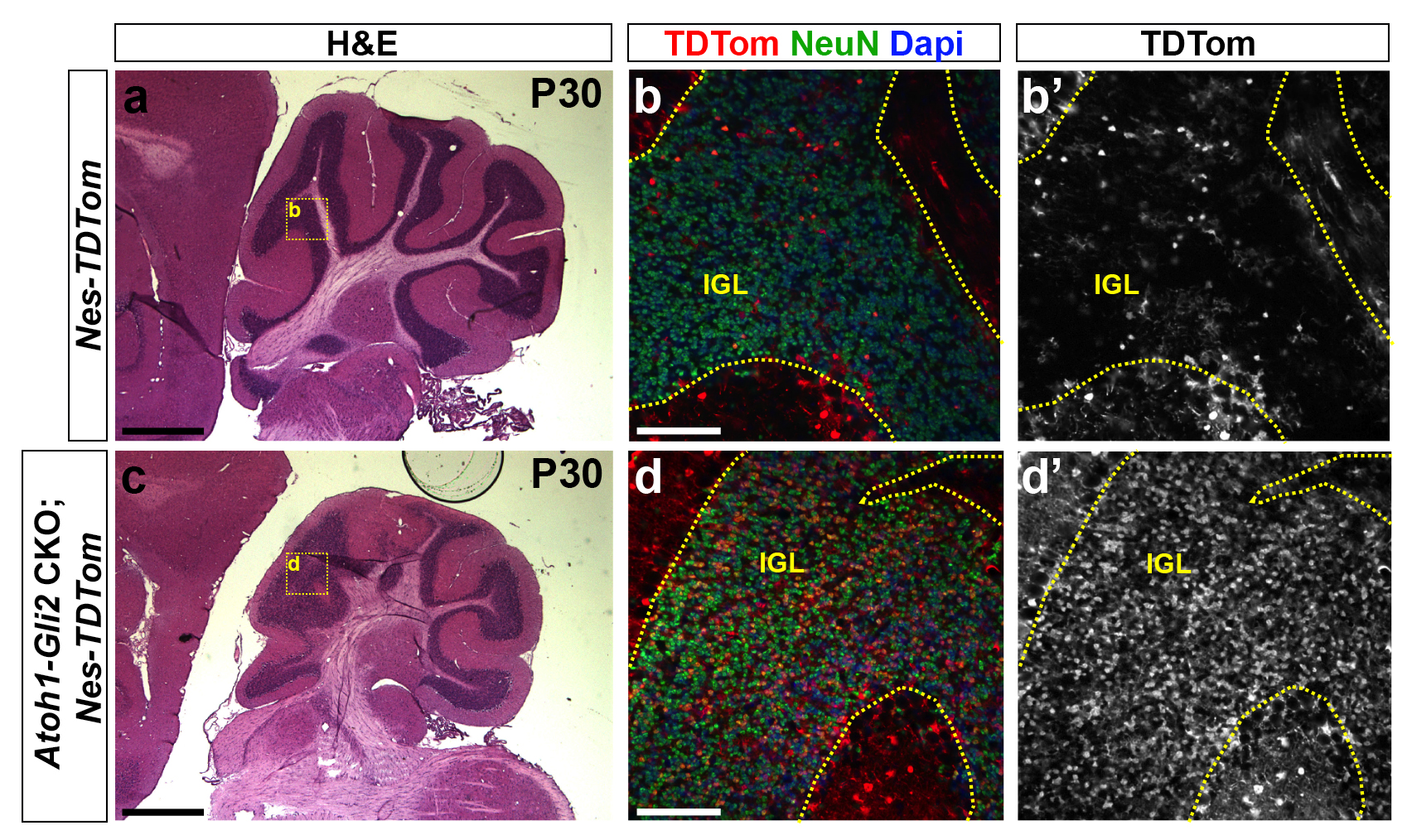

Supplement: Supplementary file 5 — Figure S4. Nestin-Expressing Progenitors (NEPs) differentiate into granule neurons in response to loss of Gli2 in the hemispheres. (a and c) H&E staining of hemispheric sagittal sections of P30 Nes-FlpoER/+; R26FSF-TDTom/+ (Nes-TDTom, a) and Atoh1-Cre/+; Gli2lox/lox; Nes-FlpoER/+; R26FSF-TDTom/+ (Atoh1-Gli2 CKO; Nes-TDTom, c) mice injected with Tm at P0. (b and d) FIHC detection of the indicated proteins and dapi on hemispheric sagittal cerebellar sections at P30. High power images are shown of the areas indicated by yellow rectangles in (a and c). IGL is indicated by the yellow doted line. Scale bars represent 1 mm (a and c) and 100 μm (b and d). (JPG 1479 kb) [file 13064_2019_128_MOESM4_ESM.jpg]

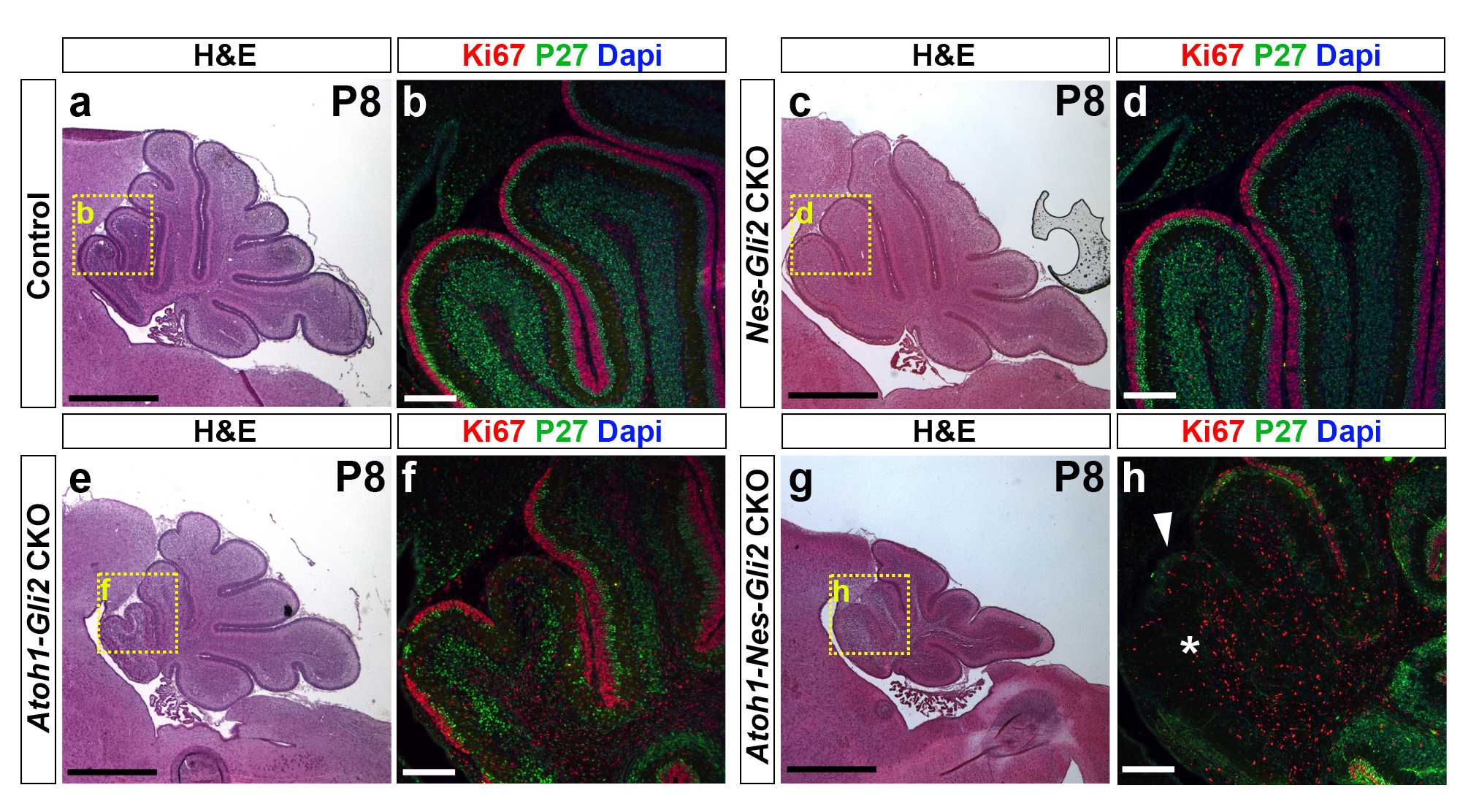

Supplement: Supplementary file 8 — Figure S5. Inactivation of Gli2 in both Nestin and Atoh1 expressing cells inhibits the recovery of the CB. (a, c, e and g) H&E staining of sagittal sections of the cerebellar vermis of P8 Gli2flox/flox (Control, a), Nes-FlpoER/+; R26MASTR/+; Gli2flox/flox (Nes-Gli2 CKO, c), Atoh1-Cre/+; Gli2flox/flox (Atoh1-Gli2 CKO, e), and Atoh1-Cre/+; Nes-FlpoER/+; R26MASTR/+; Gli2flox/flox (Atoh1-Nes-Gli2 CKO, g) mice injected with Tm at P0. Note that inactivation of Gli2 only in Nestin-expressing cells has no major effect at P8. However, inactivation of Gli2 in Nestin-expressing cells inhibits the compensation mechanism (g compared to e). (b, d, f and h) Close-up (as shown by yellow squares in a, c, e and g) of anterior vermis of P8 Gli2flox/flox (b), Nes-FlpoER/+; R26MASTR/+; Gli2flox/flox (Nes-Gli2 CKO, d), Atoh1-Cre/+; Gli2flox/flox (Atoh1-Gli2 CKO, f), and Atoh1-Cre/+; Nes-FlpoER/+; R26MASTR/+; Gli2flox/flox (Atoh1-Nes-Gli2 CKO, h) cerebella stained with the indicated proteins and dapi. White arrowhead and white asterisk indicate the loss of EGL and IGL respectively in the Atoh1-Nes-Gli2 CKO. Scale bars represent 1 mm (a, c, e and g) and 100 μm (b, d, f and h). (JPG 2032 kb) [file 13064_2019_128_MOESM8_ESM.jpg]
